# Human calmodulin mutations cause arrhythmia and affect neuronal function in *C. elegans*

**DOI:** 10.1093/hmg/ddad042

**Published:** 2023-03-15

**Authors:** Helene H Jensen, Magnus T Frantzen, Jonas L Wesseltoft, Ana-Octavia Busuioc, Katrine V Møller, Malene Brohus, Palle R Duun, Mette Nyegaard, Michael T Overgaard, Anders Olsen

**Affiliations:** Department of Chemistry and Bioscience, Aalborg University, Aalborg Ø 9220, Denmark; Department of Chemistry and Bioscience, Aalborg University, Aalborg Ø 9220, Denmark; Department of Chemistry and Bioscience, Aalborg University, Aalborg Ø 9220, Denmark; Department of Chemistry and Bioscience, Aalborg University, Aalborg Ø 9220, Denmark; Department of Chemistry and Bioscience, Aalborg University, Aalborg Ø 9220, Denmark; Department of Chemistry and Bioscience, Aalborg University, Aalborg Ø 9220, Denmark; Department of Health Science and Technology, Aalborg University, Aalborg Ø 9220, Denmark; Department of Health Science and Technology, Aalborg University, Aalborg Ø 9220, Denmark; Department of Chemistry and Bioscience, Aalborg University, Aalborg Ø 9220, Denmark; Department of Chemistry and Bioscience, Aalborg University, Aalborg Ø 9220, Denmark

## Abstract

In humans, mutations in calmodulin cause cardiac arrhythmia. These mutations disrupt the ability of calmodulin to sense calcium concentrations and correctly regulate two central calcium channels, together obstructing heart rhythm. This correlation is well established, but also surprising since calmodulin is expressed in all tissues and interacts with hundreds of proteins. Until now, most studies have focused on cardiac cell function and regulation of specific cardiac targets, and thus, potential other effects of these mutations have largely been unexplored. Here, we introduce the nematode *Caenorhabditis elegans* as an *in vivo* model to study effects of three human calmodulin mutations with different impairment on calcium binding. We find that arrhythmic effects of the calmodulin mutations N54I and D96V can be recapitulated in disruption of two rhythmic behaviors, pharynx pumping and defecation motor program. Interestingly, we also find that these mutations affect neuronal function, but in different ways. Whereas D96V sensitizes signaling at the neuromuscular junction, N54I has a protective effect. The mutation N98S did not affect rhythmic behavior, but impaired chemosensing. Therefore, pathogenic calmodulin mutations act through different mechanisms in rhythmic behavior and neuronal function in *C. elegans,* emphasizing the strength of using live multicellular models. Finally, our results support the hypothesis that human calmodulin mutations could also contribute to neurological diseases.

## Graphical Abstract

**Graphical Abstract:**
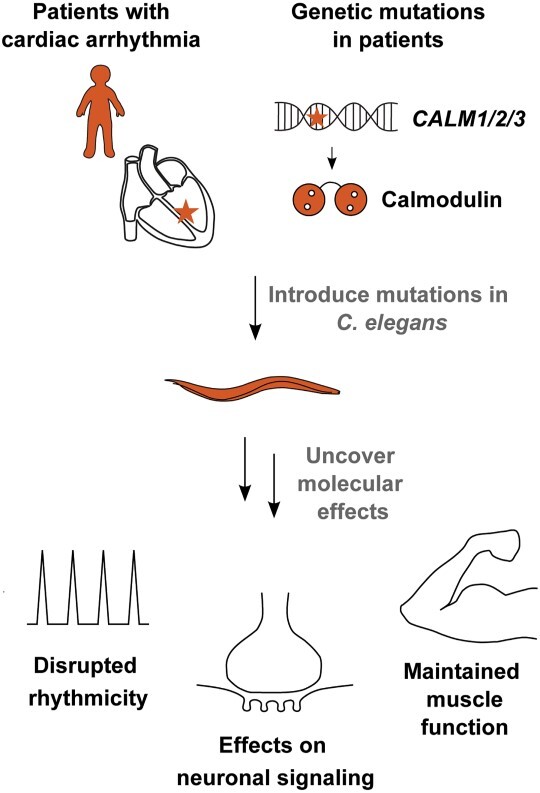

## Introduction

Across the evolutionary tree of life, calcium (Ca^2+^) signaling controls hundreds of processes including fertilization, apoptosis, cell migration, muscle contraction, vesicle release and memory formation. These finely regulated processes are managed by Ca^2+^ channels and sensor proteins that precisely relay signal information to downstream targets (1,2).

Calmodulin is a Ca^2+^ sensor that binds Ca^2+^ ions entering the cell. Calmodulin changes conformation upon Ca^2+^ binding and consequently affinity toward its more than 300 target proteins. In this way, calmodulin modulates the responses of its target proteins to the incoming Ca^2+^ signals (3,4). Calmodulin is one of the most well-conserved proteins known (5). Humans have three calmodulin genes located on separate chromosomes. They all encode the exact same protein. Before 2012, no protein altering genetic variants (mutations) within calmodulin had been reported, underscoring its extraordinary conservation. In 2012, we published the first cases of calmodulin mutations identified in patients suffering from cardiac arrhythmia (6). Since then, several reports have confirmed that, although extremely rare, mutations in calmodulin cause cardiac arrhythmias in humans, primarily catecholaminergic polymorphic ventricular tachycardia (CPVT) or long QT syndrome (LQTS) (3,7,8). In most cases, the disease is present already in childhood, in some cases even prenatally, leading to a number of tragic cardiac arrests and deaths in children.

The arrhythmogenic calmodulin missense mutations demonstrate a reduction in Ca^2+^ affinity and reduced interaction to or regulation of the voltage-gated Ca^2+^ channel isoform 1.2 (Ca_V_1.2) and/or ryanodine receptor type 2 (RyR2). Generally, we find that mutations displaying strong impairment of Ca^2+^ binding and dysregulation of Ca_V_1.2 are linked with LQTS. On the other hand, calmodulin mutations carried by individuals affected by CPVT impose dysregulation of RyR2 (9,10).

In this study, we included the mutation D96V as an example of an LQTS mutation (Table 1). This mutation was found in a child, who presented with bradycardia already during gestation and markedly prolonged QT_c_ interval from birth. In her first years of life, she suffered from cardiac arrest and numerous cases of ventricular fibrillation (7). At the protein level, the D96V mutation strongly impairs Ca^2+^ binding and Ca_V_1.2 binding and regulation. Hence, we categorize D96V as a ‘severe’ calmodulin mutation (Table 1). As an example of a CPVT mutation, we included N54I (Table 1). This mutation was found in a large family, where most carriers suffered from cardiac arrest or syncope during exercise or acute stress later in childhood (6). In contrast to D96V, the impact of N54I on Ca^2+^ binding and Ca_V_1.2 binding and regulation are very low (Table 1), and we therefore consider N54I a ‘mild’ mutation. Finally, we included N98S, which has been found in different patients, who suffer from LQTS, CPVT or a combination of these (6,8). The effects of N98S on Ca^2+^ binding and Ca_V_1.2 binding and regulation are intermediate of D96V and N54I (Table 1). It is important to stress that our mild, intermediate and severe classifications only relate directly to the impact on Ca^2+^ binding. In patients, all the mutations have a very severe impact on cardiac health.

**Table 1:** Overview of arrhythmogenic calmodulin variants

	N54I	D96V	N98S
Amino acid substitution (HGVS nomenclature)^a^	NP_008819.1:p.Asn54Ile	NP_001734.1:p.Asp96Val	NP_008819.1:p.Asn98Ser
SNP ID^a^	rs267607276	rs730882254	rs267607277
Patient diagnoses	CPVT	LQTS	CPVT/LQTS
Mutation position	N-lobe	C-lobe	C-lobe
Impact on Ca^2+^ binding	Mild	Severe	Intermediate
Impact on Ca_V_1.2 binding	Mild	Severe	Intermediate
Impact on Ca_V_1.2 regulation	Mild	Severe	Intermediate
Impact on RyR2 binding	Mild	Severe	Intermediate
Impact on RyR2 regulation	Severe	Severe	Severe
References	6,8,14,15,57–60	7,8,13,14,57,58,61	6,8,13–15,57,58,61–64

^a^We here exemplify positions of specific patient calmodulin mutations in *CALM1* or *CALM2*, but some mutations have been observed in more than one of the three genes *CALM1–3*. HGVS, Human Genome Variation Society, SNP, single nucleotide polymorphism.

Until now, most research on calmodulin mutations has been focused on cardiac mechanisms, primarily in cells, and *in vitro* studies of interactions with specific molecular targets (11–14). Given the wide span of calmodulin-target interactions, it remains a conundrum whether calmodulin mutations affect other tissues than the heart and maybe even cause other diseases. Such studies require more complex models, but few studies in animals have been conducted. Two studies found that in zebrafish, cardiac arrhythmia could be induced by calmodulin mutations N54I, N98S and D130G, but effects on other tissues were not studied (15,16). Similarly, mice carrying the mutation N98S display cardiac effects, but no other phenotypes were reported (17). Although non-cardiac phenotypes can be explored in these models, they are less suited for such studies, as they are expensive, challenging to genetically modify and limited by ethical considerations. Thus, there is a need for a system without these limitations to explore other effects of calmodulin mutations.

With this study we explore the potential of *Caenorhabditis elegans* as a model system to study calmodulin mutations. Central Ca^2+^ signaling pathways, proteins and tissues are well conserved in this nematode. *C. elegans* are small (approximately 1 mm), have a short life cycle (three days from egg to adult) and can easily be genetically modified by editing or genetic crossing to explore specific pathways (18). *C. elegans* do not have a heart, but the effects of human mutations causing CPVT and LQTS display arrhythmic pharyngeal pumping in the feeding organ (19–21). This is an important advantage, since very pathogenic mutations can be studied without causing cardiac arrest.

We hypothesized that *C. elegans* could serve as a model to study arrhythmogenic human calmodulin mutations and in addition provide a feasible platform to study how these mutations affect Ca^2+^ regulated functions in other tissues. Using CRISPR/Cas9, we designed a *C. elegans* strain expressing the human variant of calmodulin and found that the humanized strain was viable and healthy. In this strain, we introduced three different pathogenic mutations N54I, N98S and D96V identified in humans, representing mild, intermediate and severe defects on Ca^2+^ binding.

We found that the mild N54I mutation and the severe D96V mutation affected rhythmic behavior in both pharynx pumping and defecation, whereas the intermediate N98S mutation had no effects. Remarkably, N54I, D96V and N98S all affected neuronal function but in different ways, potentially affecting different targets in neurons.

## Results

**Figure 1:**
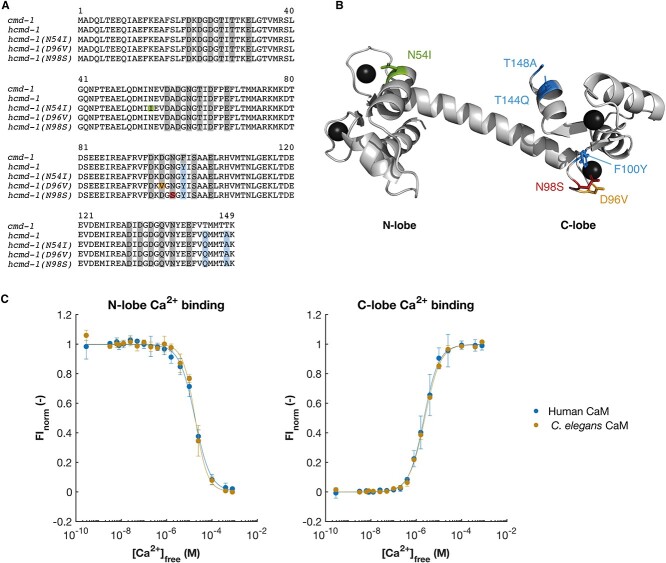
Generation of *C. elegans* strains expressing the human calmodulin protein and three cardiac mutations. (**A**) Protein sequence alignment of the calmodulin protein expressed in the *C. elegans* strains in this study: wild-type calmodulin in N2 (expressed from *cmd-1*), humanized calmodulin expressed in *hcmd-1* and three strains expressing the human calmodulin with additional arrhythmogenic mutations (*hcmd-1(N54I)*, *hcmd-1(D96V)* and *hcmd-1(N98S)*). Note that the immature numbering of calmodulin residues is used. The initial methionine is cleaved off the mature protein, in which the numbering would be N53I, D95V and N97S. Residues involved in Ca^2+^ binding are indicated in gray. Residues that differ between human and worm are indicated in blue, and positions of arrhythmia mutations are indicated in green (N54I), orange (D96V) and red (N98S). (**B**) Protein structure of human calmodulin (PDB: 1cll) in gray with four Ca^2+^ ions bound (black). Colors as in A. (**C**) Ca^2+^ binding to purified human (blue) or *C. elegans* (orange) calmodulin protein. Binding of Ca^2+^ induces a structural change in the protein that can be measured as a change in intrinsic fluorescence. *P*-values from statistical tests are given in Supplementary Material, Table S1. CaM, calmodulin, Fl_norm_, normalized fluorescence intensity.

The calmodulin proteins in *C. elegans* and humans differ in only three amino acid positions: At position 100, there is a conservative substitution from phenylalanine in *C. elegans* to tyrosine in humans (F100Y). The two other substitutions are T144Q and T148A in the C-terminal end (Fig. 1A and B). As the protein is highly conserved, we first asked if the differences between calmodulin found in humans and in *C. elegans* were detrimental for the Ca^2+^ sensing properties of calmodulin. We expressed and purified the native *C. elegans* and human protein variants and measured their Ca^2+^-binding affinities. Calmodulin has two lobes, which each bind two Ca^2+^ ions (Fig. 1B). In both lobes, we found no difference in the Ca^2+^-binding properties of the human and *C. elegans* protein variants (Fig. 1C and Table 2). We also found no difference in the Ca^2+^-dependent interaction of calmodulin to the predicted calmodulin-binding domains of the two important interaction partners, egg-laying defective 19 (EGL-19) (orthologue of Ca_V_1.2) and uncoordinated 68 (UNC-68) (orthologue of RyR2) (Supplementary Material, Fig. S1). As there were no differences in the binding properties of human and *C. elegans* calmodulin, a *C. elegans* strain expressing human calmodulin is an appropriate background to study the effects of mutations in the human calmodulin protein.

**Table 2:** Ca^2+^-binding properties of human and *C. elegans* calmodulin protein

	N-domain Dissociation Constant, *K*_D,app_ (mean ± SD, μm)	N-domain Hill Coefficient, *n* (mean ± SD)	C-domain Dissociation Constant, *K*_D,app_ (mean ± SD, μm)	C-domain Hill Coefficient, *n* (mean ± SD)
Human CaM	17.6 ± 2.9	1.30 ± 0.33	2.37 ± 0.93	1.36 ± 0.21
*C. elegans* CaM	18.52 ± 3.4	1.63 ± 0.20	2.53 ± 0.24	1.16 ± 0.15
*P*-value	0.74	0.23	0.79	0.26

The Ca^2+^ affinity measured as apparent dissociation constants, *K*_D,app,_ and Hill coefficients, *n*, for the two lobes were extracted from a Hill fit of the CaM Ca^2+^ titration data. The data were compared using Student’s *t*-test.

Whereas humans have three calmodulin-encoding genes (*CALM1-3*), *C. elegans* only expresses calmodulin from one gene (*cmd-1*). As discussed above, there are only three amino acid differences (F100Y, T144Q, T148A) in the protein produced from the *C. elegans* and the human genes. We genetically engineered *C. elegans* with CRISPR/Cas9 to create a ‘humanized’ mutant *hcmd-1(F100Y, T144Q, T148A)* (from here referred to as *hcmd-1*)*.* This ‘humanized’ *C. elegans* strain expresses the human calmodulin protein from a modified version of the endogenous *cmd-1* gene (Figs 1A, B and 7 in Materials and Methods). To examine the effect of the pathogenic mutations N54I, D96V and N98S, we used CRISPR/Cas9 to introduce them in the *hcmd-1* mutant background. These new strains are referred to as *hcmd-1(N54I), hcmd-1(D96V) and hcmd-1(N98S),* respectively (Fig. 1A, B and Table 1).

### The D96V mutation dramatically affects *C. elegans* size and fertility

**Figure 2:**
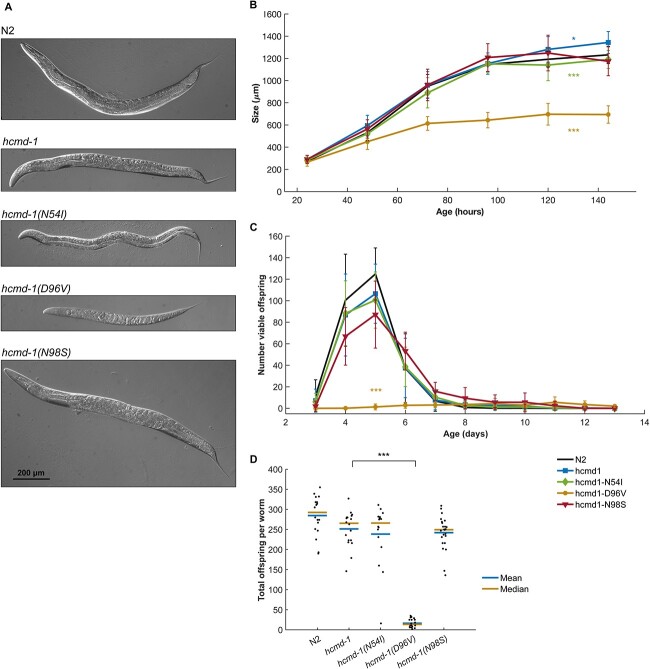
*C. elegans* carrying the D96V mutation have reduced growth and fertility. (**A**) Representative examples of four-day-old (4d) *C. elegans* worms (except *hcmd-1(D96V),* 6d) expressing the calmodulin protein variants indicated in Figure 1. (**B**) Length of worms as a function of age. Each data point corresponds to 7–69 worms, pooled from six independent experiments. Error bars show standard deviation. (**C**) Number of viable offspring per worm per day. *n* = 27–29 for each strain, pooled from three independent experiments. Error bars show standard deviation. (**D**) Total number of viable offspring per worm. *n* = 16–24 for each strain, pooled from three independent experiments. Statistical tests: B, two-way analysis of variance (ANOVA) and Tukey–Kramer post-hoc test. C, repeated measures ANOVA and Tukey–Kramer post-hoc test. D, Student’s *t*-test with Bonferroni correction. *P*-values from statistical tests are given in Supplementary Material, Table S1.

Since calmodulin is widely involved in Ca^2+^ signaling pathways in the cell, we first asked how these *hcmd-1* mutations affected general worm health. We addressed this question by quantifying the size and fertility of the worms (Fig. 2).

The *hcmd-1* mutants were slightly, but significantly, larger in size after reaching adulthood compared to wild-type N2 worms, but had otherwise overall normal morphology (Fig. 2A and B). When comparing *hcmd-1* to the three strains carrying arrhythmogenic mutations, the D96V mutation, which has the most severe impact on Ca^2+^ binding, gave the most dramatic effects. The *hcmd-1(D96V)* worms were small and carried few eggs *in utero* (Supplementary Material, Figs S2 and S3). Introduction of the mild N54I mutation conferred a small but significant reduction in size after reaching adulthood, but no other effects on morphology were observed. The *hcmd-1(N98S)* mutants had a very high number of eggs *in utero* suggesting an Egl phenotype, but in terms of size *hcmd-1(N98S)* mutants were not different from *hcmd-1* worms (Supplementary Material, Figs S2 and S3).

Humanizing calmodulin in *hcmd-1* mutants caused an insignificant reduction in viable offspring compared to the wild-type strain N2 (Fig. 2C and D). The introduction of N54I and N98S did not further affect fertility. Furthermore, the N98S mutation caused a slight but insignificant delay in egg laying (Fig. 2C). On the contrary, the D96V mutation caused a significant reduction in fertility. On average, the D96V mutants produced 93.5% fewer viable offspring than the *hcmd-1* strain. Further, most *hcmd-1(D96V)* worms had their first offspring at day 5 or 6, as opposed to day 3 or 4 for the other strains. Therefore, throughout this study we used six-day-old worms of *hcmd-1(D96V)*, whereas all other strains were used at four-day-old to match their stage of development. From differential interference contrast (DIC) microscopy (Supplementary Material, Fig. S2) and DAPI staining (Supplementary Material, Fig. S3), it was clear that *hcmd-1(D96V)* mutants had under-proliferated germlines with very few germ cells which is consistent with the very reduced fertility.

Together, these experiments showed that the humanizing genetic edits and the two arrhythmogenic mutations N54I and N98S had no or minor effects on worm fertility and growth. On the other hand, the arrhythmogenic mutation D96V had severe effects on both fertility and growth. Interestingly, this suggests that the severity of Ca^2+^-binding defects in human calmodulin variants is reflected in phenotypic strength in *C. elegans*.

### N54I and D96V mutations disrupt pharynx pumping

The pharynx is the feeding organ of *C. elegans,* and it shows many physiological and mechanical properties similar to the human heart (22,23). The pharynx is composed of 20 muscle cells, which are connected by gap junctions and controlled by a mostly autonomous nervous system. Rhythmic contractions (*pumping*) of the pharynx allow *C. elegans* to eat bacteria. In the presence of food, the pharynx pumps at a high frequency, and both human CPVT and LQTS mutations in other genes can disrupt its rhythmicity (19–21).

**Figure 3:**
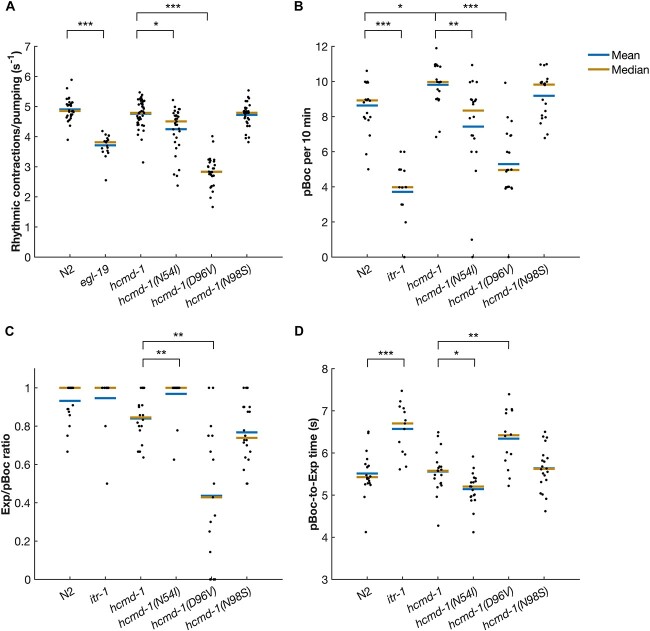
N54I and D96V mutations impair rhythmic behavior in pharynx pumping and DMP. (**A**) The number of pharynx pumps was manually counted from movies of worms feeding on OP50. *n* = 18–43, pooled from four independent experiments. (**B–D**) The two muscle contractions pBoc (P) and Exp (X) in DMP were recorded by observing actively feeding worms. *n* = 15–20, pooled from 10 independent experiments. Example ethograms from DMP experiments are shown in Supplementary Material, Figure S4. (B) Number of pBoc contractions per 10 min. (C) Calculated number of Exp contractions per pBoc per worm. (D) Average time from pBoc to Exp. Statistical tests: A–C, Wilcoxon rank-sum test with Bonferroni correction. D, Student’s *t*-test with Bonferroni correction. *P*-values from statistical tests are given in Supplementary Material, Table S1.

We quantified how the calmodulin mutations affected pharynx pumping in *C. elegans* (Fig. 3A). As positive control, the *egl-19(n2368)* mutant was included. This mutant carries a point mutation in the voltage-gated Ca^2+^ channel EGL-19. The *n2368* allele is a known LQTS mutation in the orthologous human protein Ca_V_1.2 (21,24,25).

The humanizing genetic edits in calmodulin did not affect pharynx pumping rate, and neither did the N98S mutation (Fig. 3A). On the other hand, the mild N54I mutation caused a significant 10.8% reduction in pumping rate compared to *hcmd-1*. The severe D96V mutation reduced the pumping rate by 40.5%, which was more than the *egl-19(n2368)* LQTS positive control. Thus, N54I and D96V both affected pharynx pumping rate in *C. elegans,* despite having different Ca^2+^-binding properties and being located in the N- and C-terminal lobes, respectively.

### N54I and D96V mutations disrupt rhythmical behavior in the defecation motor program

Motivated by the effects on pharynx pumping and the difference in impact from the N54I and D96V mutations, we turned our attention to another rhythmic behavior in *C. elegans*, namely the defecation motor program (DMP), which is regulated by Ca^2+^ signaling (26). This autonomous motor program is activated approximately once every minute in actively feeding worms and consists of a distinct set of muscle contractions (27). The DMP cycle is initiated by an easily recognizable contraction in the posterior body-wall muscles (pBoc, P). This is followed by a smaller contraction in the anterior body-wall muscles (aBoc). Finally, the gut content is expelled by a characteristic contraction of enteric muscles—referred to as Expulsion (Exp, X). Knock-down studies using RNAi against calmodulin in the intestine have shown titratable effects on the DMP cycle regulation, accompanied by disrupted Ca^2+^ signals (28).

Several genes are known to regulate specific steps of the DMP cycle. Here, we included the *itr-1(sa73)* mutant strain as a positive control. These mutants carry a mutation in the nematode IP_3_ receptor, which is a Ca^2+^ channel and a main initiator of the DMP cycle. We find that *itr-1(sa73)* mutants initiate fewer DMP cycles (Fig. 3B) as previously reported (29).

The humanizing mutations caused a small but significant increase in cycle frequency compared to N2 (Fig. 3B and Supplementary Material, Fig. S4). Like for the rate of pharynx pumping, we found a reduction in DMP cycle frequency for *hcmd-1(N54I)* and *hcmd-1(D96V)* mutants compared to *hcmd-1*, measured as the number of pBoc contractions per 10 min (Fig. 3B and Supplementary Material, Fig. S4). The N98S mutation did not have any effects on DMP cycle frequency.

We then asked whether these mutations affected the ability of the worms to complete the full motor program by quantifying the number of finalized Exp events relative to initiated pBoc events (Fig. 3C). The *hcmd-1(D96V)* mutant frequently failed to complete the DMP cycle (43.7% complete cycles). Conversely, *hcmd-1(N54I)* mutants displayed significantly *more* complete DMP cycles (96.9%) compared to the *hcmd-1* controls (84.0%). Finally, we quantified the time interval between pBoc and Exp contractions (Fig. 3D). The D96V mutation caused a 14.0% increase in pBoc-to-Exp time, whereas the N54I mutants had reduced time interval by 7.5% compared to *hcmd-1* controls.

In summary, we found that the humanizing edits and the N98S mutation had no or small effects on DMP regulation. On the other hand, regulation of DMP cycle initiation and termination was impaired in *hcmd-1(D96V)* mutants. The N54I mutation caused a reduction in the initiation frequency of the DMP cycle, but a small increase in termination efficiency.

### Rhythmic behaviors are not altered by calmodulin mutations due to a systemic disruption of muscle function

Together, the data presented above suggest that the arrhythmogenic calmodulin mutations N54I and D96V, but not N98S, affect rhythmicity in *C. elegans*. Since calmodulin is involved in many Ca^2+^ signaling pathways and thus is key in regulating muscle function, we speculated whether these effects could result from a general impairment of muscle function.

**Figure 4:**
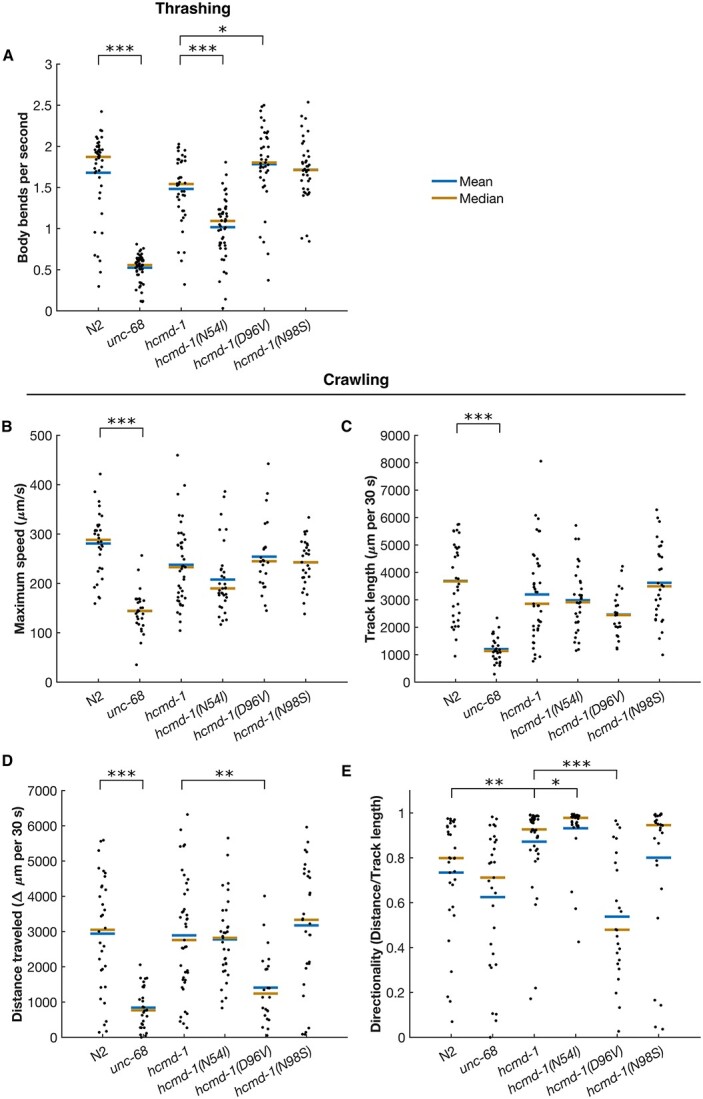
N54I and D96V have opposite effects on worm motility. (**A**) Average speed of body bends during swimming/thrashing. *n* = 39–53, pooled from five independent experiments. (**B–E**) Motility of worms on plates. *n* = 24–41, pooled from four individual experiments. (B) Maximum speed. (C) Total length of track in 30 s (speed). (D) Net distance traveled from start to end in 30 s. (E) Directionality measured as the net distance traveled relative to the total track length. Statistical tests: A**–**B, Student’s *t*-test with Bonferroni correction. C**–**E, Wilcoxon rank-sum test with Bonferroni correction. *P*-values from statistical tests are given in Supplementary Material, Table S1.

To answer this question, we turned our attention to motility as a general measure of muscle function. We measured the body bend frequency (thrashing) during swimming (Fig. 4A). As positive control, we included the slowly moving *unc-68(r1162)* strain, which expresses a loss-of-function version of the Ca^2+^ channel UNC-68, the nematode ryanodine receptor. We did not see any effects on thrashing frequency by the humanizing edits or the N98S mutation. The N54I mutation decreased thrashing frequency by 31.4%, while the D96V mutation resulted in a 20.3% increase, compared to *hcmd-1* (Fig. 4A). It is intriguing that two arrhythmogenic mutations had opposite effects and that mutants harboring the severe D96V mutation displayed increased thrashing frequency. Therefore, we quantified maximum speed of worms moving on nematode growth medium (NGM) plates with food, and we found the same trends as for thrashing, however not statistically significant (Fig. 4B). When assessing the average speed measured as the track length moved in 30 s, we did not find any significant effects of the genetic modifications (Fig. 4C).

Finally, we asked if the crawling worms moved directly forward, or if they turned or reversed during movement. Thus, we quantified the net distance traveled from start to end of the 30 s migration. As a measure of directionality, we calculated the ratio between the net distance moved and the track length for each worm (Fig. 4D and E) (30). We found that the humanizing genetic edits caused a significant 10.6% increase in directionality, which was further increased by the N54I mutation by 6.7%. N98S gave an insignificant 8.4% decrease, whereas the D96V mutation had the strongest effect on directionality, showing a 38.4% reduction compared to *hcmd-1*.

In summary, we found that both the humanizing genetic edits and that the N54I and D96V mutations affected motility, the latter two in opposite directions. We however note that all strains were motile, implying that disruption of rhythmicity cannot alone be explained by a general impairment of muscle function.

### Arrhythmogenic mutations influence neuronal function

We found that N54I and D96V had opposite effects on DMP termination and directionality during movement. These functions are under neuronal regulation (27,31,32). Calmodulin is expressed in neurons (33), and Ca^2+^ signaling plays an important role in the release of neurotransmitters and proper neurotransmission (34). Therefore, we hypothesized that the calmodulin mutations could affect the function of neurons innervating muscles involved in rhythmic behavior.

**Figure 5:**
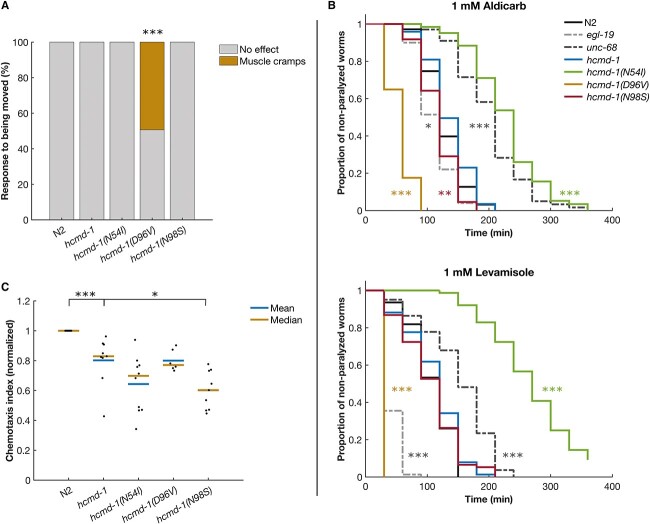
*C. elegans* with the D96V calmodulin mutation are hypersensitive to aldicarb and levamisole, whereas N54I carriers are resistant. (**A**) Systemic full body convulsions when transferring worms with platinum picker. *n* = 75 for each strain. See Supplementary Material, Movies S1 and S2 for an example. (**B**) Worms were placed on NGM plates with 1 mm aldicarb (top) or levamisole (bottom). Every 30 min, paralyzed worms were counted and removed. *n* = 57–79, pooled from three independent experiments. Asterisks in gray indicate differences from N2. Asterisks in color indicate differences from *hcmd-1*. (**C**) Worms were placed on plates with drops of the chemoattractant benzaldehyde or a control buffer. A chemotaxis index of 1 indicates that all worms moved to the chemoattractant. A chemotaxis index of 0 indicates random movement. *n* = 6–11 experiments combining a total of 211–1463 worms. Statistical tests: A, Fisher’s Exact test. B, Bonferroni-corrected log rank test. C, Bonferroni-corrected Wilcoxon rank-sum test. *P*-values from statistical tests are given in Supplementary Material, Table S1.

Interestingly, during maintenance of the worms, we observed that *hcmd-1(D96V)* exhibited systemic muscle cramps or full body convulsions (Fig. 5A, Supplementary Material, Movies S1 and S2). These convulsions were induced by moving the worms with a platinum picker between NGM plates during routine maintenance. Furthermore, we observed that when moving the same worm repeatedly, the convulsions increased in frequency and duration (data not shown). Since similar convulsions have been reported as a result of defective neurotransmission (35), we speculated that the calmodulin mutations negatively affect neuromuscular signaling.

We addressed this using a well-established paralysis assay with aldicarb and levamisole to identify presynaptic (neuronal) and postsynaptic (muscular) effects, respectively. These drugs target cholinergic signaling at the neuromuscular junction (36,37). As controls, we again included *egl-19(n2368)* and *unc-68(r1162)*, as *egl-19* and *unc-68* both affect cholinergic signaling (24,38,39). Aldicarb (a cholinesterase inhibitor) causes accumulation of acetylcholine in the synapse. If a mutation affects the efficiency of neurotransmitter release, paralysis will happen faster or slower. Consistent with previous studies, the *unc-68* null allele was hyposensitive to aldicarb (38), and the LQTS *egl-19* allele was slightly, but significantly, hypersensitive (Fig. 5B, upper panel). Interestingly, we found that *hcmd-1(N54I)* were hyposensitive to aldicarb, whereas *hcmd-1(D96V)* were hypersensitive. There was a small although significant increase in sensitivity for *hcmd-1(N98S)* as well (Fig. 5B, upper panel).

Levamisole (a nicotinic acetylcholine receptor agonist) directly activates the acetylcholine receptor causing continued muscle stimulation and therefore identifies postsynaptic effects (40). We found a protective effect of N54I and increased sensitivity in the *hcmd-1(D96V)* mutant, indicating that these mutations also affect acetylcholine reception and/or its downstream targets, again in opposite directions (Fig. 5B, lower panel). This is consistent with calmodulin having both pre- and postsynaptic effects and importantly shows that these functions can be separated by specific mutations in calmodulin.

To determine if the presynaptic effects of calmodulin mutations would manifest in other neuronal defects, we examined their effect on chemotaxis. Here, worms are allowed to move freely on a plate prepared with two drops of benzaldehyde and two drops of a control buffer in opposite quadrants and with equal distance to the center of the plate. Benzaldehyde is a chemoattractant sensed by the AWC neurons in *C. elegans* (41). Azide was applied to each spot to paralyze the worms upon arrival at the test or control spots. After 60 min, we found that almost all N2 controls had found their way to the chemoattractant. The *hcmd-1* worms were 19.8% less efficient in sensing the chemoattractant compared to N2 (Fig. 5C). Chemotaxis was further reduced in the *hcmd-1(N54I)* and *hcmd-1(N98S)* mutants by 19.8% and 25.0%, respectively. Remarkably, despite that D96V has the most severe Ca^2+^-binding defect, the chemotaxis index of the *hcmd-1(D96V)* mutants was not different compared to *hcmd-1*.

Together, we find neuronal effects of arrhythmogenic calmodulin mutations in *C. elegans*. Briefly, D96V strongly attenuates signaling at the neuromuscular junction. N54I has opposite effects to D96V at the neuromuscular junction. Although *hcmd-1(N98S)* has shown no or weak effects in rhythmic behavior and motility, we find that thus mutant shows the strongest chemotaxis defect.

## Discussion

Calmodulin is extremely conserved across species but not trivial to study in humans where carriers of calmodulin mutations are exceedingly rare and deadly. Furthermore, calmodulin is highly pleiotropic which adds to the complexity of studying calmodulin variants *in vivo,* but at the same time stresses the need for using model organisms. To overcome these obstacles, we have established *C. elegans* as a model to study human calmodulin mutations. In *C. elegans*, the calmodulin gene *cmd-1* is essential and RNAi results in embryonic lethality (42). Therefore, the role of calmodulin in *C. elegans* is largely underexplored, and no mutation has yet been characterized. The *C. elegans* calmodulin protein differs from human calmodulin only in three amino acids, and we find that the two proteins have similar Ca^2+^ and target-binding properties *in vitro*. Consistent with this, humanizing the native *C. elegans* calmodulin *in vivo* does not cause any strong phenotypes across the investigated traits. This is in stark contrast to the introduction of calmodulin variants known to affect Ca^2+^ binding and to cause cardiac arrhythmia in humans, which cause a range of phenotypes in the worms.

### *C. elegans* as a model of arrhythmia induced by human calmodulin mutations

**Figure 6:**
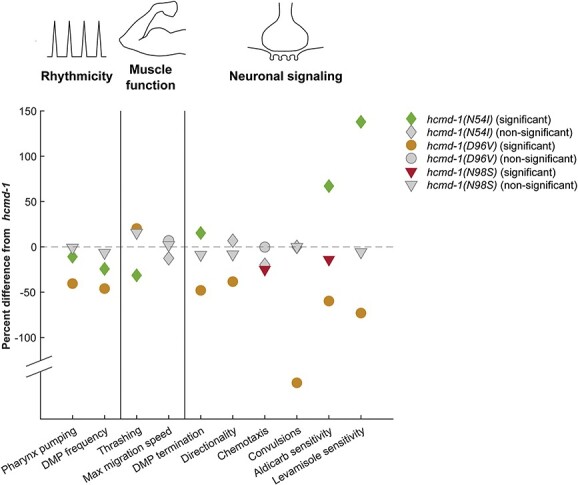
Data summary. Mean values for indicated experiments were extracted, and the difference from *hcmd-1* was calculated. For convulsions, the results are shown as ‘lack of convulsions’ to reflect the negative effects of calmodulin mutations. For the paralysis assays, the mean paralysis time was used to calculate the difference. Color-filled symbols indicate statistical significance in the figures presented.

Together, we find that the arrhythmogenic effect of the mutations N54I and D96V, but not N98S, can be recapitulated in assays of pharynx pumping and DMP in *C. elegans* (Fig. 6).

The CPVT-mutation N54I has mild or no effects on Ca^2+^ binding, and it is one of the only mutations found in the N-terminal lobe of the protein (3,6,8). N54I can induce arrhythmia in zebrafish (15). In this study, we found significant effects on rhythmical behavior in *C. elegans*, but the animals were otherwise healthy with normal morphology, fertility and development.

On the contrary, the LQTS-mutation D96V is one of the most severe calmodulin mutations, manifesting already prenatally (7). In *C. elegans*, D96V also manifested severely, resulting in animals that could only just reproduce. The mutation had strong effects on rhythmical behavior, correlating well with the phenotype found in humans. *hcmd-1(D96V)* worms were fully motile with a slight increase in speed compared to controls. This intact muscle function is important to note as it demonstrates that D96V does not systemically disrupt all Ca^2+^ signaling in *C. elegans*.

This study did not find arrhythmia effects of N98S in humanized *C. elegans*. Interestingly, this mutation has been found in several patients, who have been diagnosed with CPVT, LQTS or a combination of the two (6,8). Due to this incongruity, the N98S mutation has been modeled in mice, which did demonstrate arrhythmic effects, however not very dramatic (17). Whether our results are a consequence of the N98S mutation having a special interaction pattern generally or because it does not affect rhythmical behavior in nematodes specifically, remains to be shown. Other studies also find that not all human arrhythmia mutations cause arrhythmia in *C. elegans* (19,20).

Although biochemically very different, both N54I and D96V mutations affect worm rhythmicity. N54I is located in the N-terminal lobe and is not involved in Ca^2+^ binding. In contrast, D96V strongly affects a central Ca^2+^-binding residue in the C-terminal lobe. These observations emphasize the importance of using both *in vitro* and *in vivo* approaches to study human disease, as they contribute complementing observations. Moreover, it displays the strength of using simple model organisms as the phenotypic differences observed could not have been predicted solely based upon Ca^2+^ binding.

### Calmodulin mutations impair neuronal function in *C. elegans*

Several previous studies have found that calmodulin is important for neuronal development and function in both *C. elegans*, *Drosophila* and mice (33,43,44). A robust association between neuronal effects and arrhythmogenic calmodulin mutations has, however, never been demonstrated. We find different neuronal effects for N54I, D96V and N98S. The mutation D96V increases aldicarb/levamisole sensitivity and decreases directional movement and DMP termination efficiency, which are also regulated by neurons (Fig. 6). Interestingly, the N54I mutation displays an opposite pattern, as it gives resistance to aldicarb and levamisole and increases DMP termination efficiency. We propose that these two mutations cause similar effects on rhythmic behavior but disrupt neuronal function through different downstream mechanisms. For example, D96V may affect a range of targets through disrupted Ca^2+^ sensing, while N54I may affect specific targets that depend on biochemical interactions with this specific residue.

Supporting this notion, only the *hcmd-1(D96V)* mutants display whole body convulsions after being transferred between plates. Similar convulsions have previously been reported following treatment of uncoordinated-43 (*unc-43*) null mutants with the γ-aminobutyric acid (GABA) receptor antagonist pentylenetetrazole (35). *unc-43* encodes a homolog of the Ca^2+^/calmodulin-dependent protein kinase II (CaMKII) which is considered a general regulator of synaptic plasticity (35,45). There are few studies available on the effects of arrhythmogenic calmodulin mutations on CaMKII, and the results are inconsistent (16,46,47). Therefore, further studies are required to elucidate whether *hcmd-1(D96V)* mutants experience whole body convulsions due to defective signaling via *unc-43* or GABAergic signaling.

The difference between D96V and N54I in aldicarb and levamisole resistance is interesting. We speculate that N54I causes resistance toward chronic excitation of neurons. Such a protective effect is supported by a recent study showing that the decline of neuronal plasticity of O_2_-sensing neurons (URX, AQR and PQR) with age is mediated via calmodulin in *C. elegans* (48). This study finds that knock down of calmodulin using cell-specific RNAi can counteract plasticity decline observed with age. We propose that a similar mechanism could be at play for the N54I mutation which may not interact correctly with specific targets and in this regard mimics a calmodulin knock down. The aldicarb/levamisole sensitivity observed for the severe D96V mutation could be due to a near complete and systemic disruption of Ca^2+^/calmodulin signaling. Alternatively, this mutation may affect other targets than those affected by the N54I mutation, such as isoforms of voltage-gated Ca^2+^ channels, which are also affected in the heart (14) or *unc-43*/CaMKII and/or the GABA signaling pathway as discussed above.

That different downstream molecular mechanisms are influenced by the calmodulin mutations is further supported by the chemotaxis analysis. Surprisingly, the D96V mutation with strongest impact on Ca^2+^ did not affect chemosensing. The neurological effects of D96V are therefore not systemic. On the contrary, the N98S mutation significantly impaired chemotaxis and aldicarb sensitivity but had no or minor effects on rhythmicity and motility. In chemotaxis, we also found that the humanizing edits in themselves significantly impacted benzaldehyde sensing. Benzaldehyde is one of several chemoattractants used in *C. elegans* (49). Whether the effects of the calmodulin mutations on chemotaxis could be chemoattractant specific remains to be shown. Together these observations support the hypothesis that calmodulin mutations can impact neuronal function, and that different calmodulin mutations can affect different downstream pathways in neurons.

There are several other assays to study neuronal regulated behavior in worms. *C. elegans* display an interesting phenotype called social feeding where members of a population aggregate on the bacterial lawn (50). Social feeding is considered a model of psychiatric illness, and many antipsychotic drugs inhibit social feeding (51). Interestingly, RNAi mediated knock down of calmodulin also inhibits social feeding, further supporting an important neuronal role of calmodulin.

### Implications for understanding human calmodulin mutations

Human calmodulin mutations are extremely rare. New patient carriers are collected in the International Calmodulinopathy Registry (8), which in 2019 reported 74 subjects, of whom 86.5% suffered from cardiac arrhythmia. Interestingly, 13 of these subjects also showed mild-to-severe neurological impairment. Whether these neurological effects were consequences of life-threatening cardiac events early in life is unknown. With the data presented here in mind, the hypothesis that calmodulin mutations could have neurological consequences in humans seems very likely.

In this study, we establish *C. elegans* as a model of cardiac arrhythmia induced by human calmodulin mutations. We demonstrate that the three arrhythmogenic mutations N54I, D96V and N98S have neurological effects, likely through different mechanisms. We thus suggest that neurological consequences of calmodulin mutations will originate from different molecular pathways, and their severity may not be predictable from what we know about calmodulin mutations today. These results are new to the domain of calmodulin mutations and underscore how animal models can contribute new and more complex information. Calmodulin is highly pleiotropic, but the fact that the studied human calmodulin mutations display separable phenotypes is promising from a drug development point of view. Future studies into underlying mechanisms have potential to form the basis for designing highly specific drug interventions impacting specific downstream pathways.

## Materials and Methods

### *C. elegans* strains and maintenance

**Figure 7:**
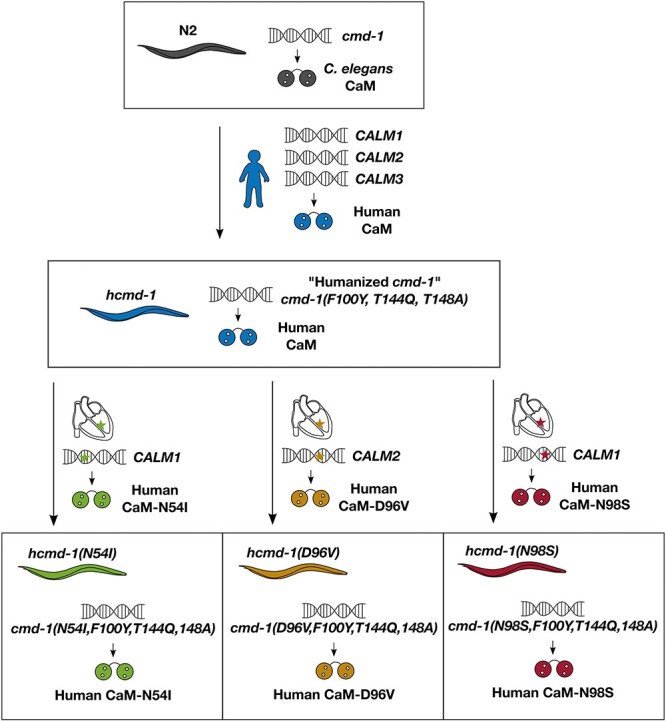
The calmodulin gene *cmd-1* in wild-type N2 was modified to express the human form of calmodulin, before introduction of an additional three arrhythmogenic mutations. There are three amino acid differences between human and *C. elegans* calmodulin protein. In *hcmd-1*, the endogenous *cmd-1* calmodulin gene was modified using CRISPR/Cas9 to express the human form of the protein. Afterwards, three different edits were further introduced to generate three different strains expressing calmodulin with amino acid substitutions identical to those found in arrhythmia patients.

The wild-type N2 strain was used as basis for generation of the *hcmd-1* strains (Fig. 7): OLS160 *hcmd-1(aar160[F100Y, T144Q, T148A])* referred to as *hcmd-1*, OLS178 *hcmd-1(aar178[N54I]) referred to as hcmd-1(N541)*, OLS183 *hcmd-1(aar183[(D96V]) referred to as hcmd-1(D96V)* and OLS171 *hcmd-1(aar171[(N98S]) referred to as hcmd-1(N98S)*. The strains JT73 *itr-1(sa73)*, MT6129 *egl-19(n2368)* and TR2171 *unc-68(r1162)* were purchased from the *Caenorhabditis* Genetic Center (CGC, Minnesota, USA).

All strains were maintained on NGM plates at 20°C. NGM plates were prepared from 3 g/L NaCl, 2.5 g/L soy peptone, 17 g/L agar, supplemented with 5 mg/mL cholesterol, 1 mm CaCl_2_, 1 mm MgSO_4_ and 25 mm potassium phosphate buffer (stock is 132 mm K_2_HPO_4_, 868 mm KH_2_PO_4_, (pH 6)) and spotted with OP50 *Escherichia coli* grown over-night in LB medium (10 g/L NaCl, 10 g/L soy peptone, 5 g/L agar, pH 7.5). For maintenance, eggs were transferred to fresh plates 1–2 times per week. Unless otherwise stated, worms were used in experiments as four-day-old adults, except *hcmd-1(D96V)*, which were six days old.

### CRISPR/Cas9 Oligos

crRNAs and single-stranded oligodeoxynucleotides (ssODNs) were designed in the online software Benchling (https://benchling.com) and ordered with PAGE purification from Sigma-Aldrich. crRNAs cleaving >10 bases away from the edit site were chosen when possible. ssODNs were made with 35–40 bp homology arms, and for silent mutations codon usage was accounted for (52).

Trans-activating CRISPR (tracr)RNA, guide CRISPR (cr)RNAs and ssODN repair templates were dissolved in TRIS buffer and kept at −20°C. Sequences and stock concentrations can be found in Supplementary Material, Tables S2 and S3.

### CRISPR/Cas9 injection protocol

The humanized calmodulin worm strain *hcmd-1* and the following disease mutants were made using the *dpy-10* co-CRISPR strategy described by Arribere and colleagues (53) and the direct delivery of *in vitro*-synthesized and -assembled Cas9-crRNA-tracrRNA complexes described by Paix and colleagues (54). For all injections, the 10 μL injection mixture was prepared as described (54), with modified concentrations. 15 μm tracrRNA, 13 μm crRNA-X and 2 μm  *dpy-10*(cn64) were mixed and placed in a thermocycler (5 min at 95°C, 5 min at 21°C). 15 μm Cas9 (produced in-house) was added and left at room temperature for 5 min. Last, ssODN-X, ssODN *dpy-10*(cn64) and nuclease-free H_2_O up to 10 μL was added. The injection mix was spun down at 13200 rpm for 4 min at 4°C, incubated at 37°C for 30 min and spun down again for 10 min. N2 worms were injected to make the *hcmd-1* strain which was backcrossed against N2 before introducing the disease mutations (N54I, N98S and D96V). Disease mutants were backcrossed three times to *hcmd-1*.

### Microinjection needles

Needles for microinjection were made by pulling Borosilicate Glass Capillaries (1B110F-4) (World precision instruments) using Narishige’s PC-100 micropipette puller with heater 1 set at 58 and no setting for heater 2. Injections were made using a FemtoJet® 4× (Eppendorf) with an injection pressure of 200 hPa.

### Mounting *C. elegans* for injections

Young adult worms were transferred to a droplet of Halocarbon oil on a glass slide with a 2% dried agarose pad. DIC on a Leica DNI300B microscope was applied for visualization of animal gonads. Animals were recovered in S-basal buffer (100 mm NaCl, 50 mm KH_2_PO_4_) and one to three animals were placed on each NGM plate to lay eggs for four days at 16°C.

### Screening for transgenes and mutants

F1 animals on jackpot brood plates (54) were screened by identifying individuals with a roller phenotype (53). Briefly, rollers were singled out and tested by polymerase chain reaction (PCR) after egg lay to identify individuals with the desired edit, and F2 wild-type worms from positive mothers were then singled out and tested by PCR to identify homozygote animals of the desired edit. Finally, the CRISPR edits were verified by Illumina sequencing.

### Genotyping of *C. elegans* stains

During gene editing work and before every experiment, correct identity of worms was ensured by PCR (N2, TR2171 and *hcmd-1* strains) or by visual inspection/quantification of characteristic traits. For PCR, 1–10 worms were frozen in lysis buffer (50 mm KCl, 10 mm Tris (pH 8.3), 2.5 mm MgCl_2_, 0.45% NP-40, 0.45% Tween-20, 0.01% (w/v) gelatin) at -80°C for at least 30 min. The lysates were then heated to 60°C for 60 min, followed by 95°C for 15 min. PCR reactions were prepared using primers indicated in Supplementary Material, Table S4 in a DreamTaq polymerase master mix (Thermo Scientific, K9022), following the manufacturer’s instructions. The PCR reactions were then analyzed in 1% or 2% agarose gels stained with Sybr Safe (Invitrogen, S33102) and developed on a ChemiDoc MP imager (Bio-Rad).

### Fertility assay

To measure the number of viable offspring for each worm, eggs of each strain were placed on separate NGM plates. After 3 days and subsequently every 24 h, the now adult worms were moved to new plates. The plates that worms were removed from were returned to the incubator for another two days to allow the offspring to develop to large larvae, easing subsequent quantification. Plates were then removed from the incubator, and the offspring was counted. In some cases, plates were stored at 4°C before counting the offspring.

### *C. elegans* size measurements

Adult hermaphrodites were placed on fresh NGM plates and allowed to lay eggs for 2 h. The adults were then removed. The offspring was imaged at 24 h intervals on an Olympus SZ61 stereomicroscope with an ANDOR Zyla 4.2 sCMOS camera and KL 300 LED lamp, managed by NIS Elements software. The length of each worm was analyzed using the Segmented Line tool in Fiji ImageJ.

### Muscle convulsion assay

Adult worms were transferred to fresh NGM plates. Worms from each strain were then moved one at a time to another place on the plate using a platinum picker in the same way as during routine maintenance. After the worm had been placed on the plate, it was observed whether it began normal movement or convulsed. The experiment was blinded and performed by two different individuals.

### DMP assay

Adult worms were moved to a fresh NGM plate. Each worm was followed for 10 min while feeding on OP50 and then sacrificed. If the worm moved away from the bacterial lawn, the experiment was stopped. pBoc and Exp muscle contractions were recorded using custom-written software. aBoc contractions were not recorded as they can easily be overlooked. The experiment was blinded.

### Pharynx pump assay

Adult worms were imaged for 15–20 s on an Olympus SZ61 stereomicroscope equipped with an ANDOR Zyla 4.2 sCMOS camera and KL 300 LED lamp, managed by NIS Elements software. The number of pumps was manually counted using the Cell counter tool in Fiji ImageJ.

### Thrashing

Adult worms were transferred to droplets of 100 μL S-basal (100 mm NaCl, 50 mm KH_2_PO_4_ (pH 6)), avoiding clumps of bacteria in the suspension. The worms were imaged for 30 s at 15 fps on an Olympus SZX16 stereomicroscope equipped with a DP72 camera and managed with Olympus CellB software.

### Motility on plates

NGM plates with thin, homogeneous OP50 lawns were generated by evenly distributing four to five drops of an OP50 over-night culture on NGM plates 12–16 h before the experiment. Adult worms were placed in a corner of the plate and allowed to move away from this region for at least 15 min. The worms were imaged for at 15 fps on an Olympus SZX16 stereomicroscope equipped with a DP72 camera and managed by Olympus CellB software.

### Imaging of *C. elegans* morphology

Adult worms were placed on agarose pads with a 10 μL drop of S-basal and covered with a coverslip. The worms were imaged on an Olympus IX83 inverted microscope equipped with a Hamamatsu Orca-Flash 4.0 camera, using a 10× UPLSAPO objective and managed by Olympus CellSens software. For DAPI staining, worms were fixed in ice-cold methanol for 5 min before being mounted in EverBrite™ Hardset Mounting medium (Biotium 23004).

### Paralysis assay

NGM plates containing either aldicarb (Sigma-Aldrich, 33386) or levamisole (MP Biomedicals, 155228) were prepared by first making 100 mm stock solutions in 70% ethanol (aldicarb) or H_2_O (levamisole). The stock solutions were added to NGM medium directly before casting plates to reach a final concentration of 1 mm. 30 μL over-night OP50 bacterial culture was added 30 min prior to the start of the assay. 20–25 age-matched worms were transferred to the drug-containing plates. Every 30 min the worms were poked up to three times with a platinum picker. If this did not provoke movement and there was no visible pharynx pumping, the worms were scored as paralyzed and removed.

### Chemotaxis assay

5 cm NGM plates were marked into four equal quadrants with a 1 cm radius circle made with origo at the center of the plate. Each pair of opposite quadrants was labelled C (control) or T (test) and a test-spot was marked in the center of the quadrant, at least 2 cm from the center circle. To this, thrice washed adult worms were applied to the center. Simultaneously, 1 μL 1 M NaN_3_ was applied to the test-spot in all quadrants along with 1 μL 1:200 benzaldehyde in EtOH (B1334, Sigma-Aldrich) in the T quadrants and 1 μL EtOH in C quadrants. After 60 min, the number of worms in each quadrant was manually counted.

### Calmodulin protein expression and purification

Full-length human and *C. elegans* calmodulin were expressed in *E. coli* Rosetta (DE3), both from a modified pET vector containing an N-terminal maltose-binding protein (MBP) and a tobacco etch virus (TEV) cleavage site. The MBP–TEV–calmodulin fusion protein was purified on an amylose affinity column (New England Biolabs) and subsequently cleaved by a TEV protease. Following cleavage, calmodulin, TEV and MBP were separated by anion ion exchange chromatography using a Q-sepharose column (GE Healthcare). The calmodulin-containing fractions were pooled and concentrated before removing residual Ca^2+^ by adding 20 mm ethylenediaminetetraacetic acid (EDTA) to the sample prior to applying it to a Superdex 75 size exclusion column (GE Healthcare). The identity and integrity of each protein preparation was confirmed by matrix-assisted laser desorption/ionization-time of flight mass spectrometry. Protein concentrations were determined by absorption at 280 nm.

### Calmodulin Ca ^2+^-binding affinity

Ca^2+^ titrations were done as previously described (9). Briefly, this assay was based on measuring intrinsic calmodulin fluorescence using a spectrofluorometer (HORIBA Jobin Yvon, FluoroMax-4P). In preparation for this, discontinuous titration points of different free Ca^2+^ concentrations, [Ca^2+^]_free_, were prepared and added 30 μm calmodulin before sequentially transferring each solution to a 3 by 3 mm cuvette for fluorescence emission spectra recording. Measurements were done in triplicate.

Ca^2+^-binding curves for the N- and C-lobes of calmodulin were extracted from the fluorescence intensity (FI) signals from Phe and Tyr emission at 280 and 310 nm, respectively, and the data were fitted according to the generic Hill equation:


$$\varTheta =a\cdot \frac{1}{1+{\left(\frac{K_{\mathrm{D},\mathrm{app}}}{{\left[{\mathrm{Ca}}^{2+}\right]}_{\mathrm{free}}}\right)}^n}+b$$

where *b* and *a* indicate the initial FI-plateau and the span in FI from low to high [Ca^2+^]_free_, respectively. *n* is the Hill coefficient and *K*_D,app_ is the apparent Ca^2+^ dissociation constant. Curve fitting was done to the raw FI data using GraphPad Prism 8.4.2, whereas data normalization was only done for visualization purposes.

### Calmodulin-binding affinity to EGL-19 and UNC-68 peptides

A two-dimensional titration assay was used to determine the affinity of human and *C. elegans* calmodulin for binding to EGL-19 and UNC-68 peptides at 4 discrete [Ca^2+^]. Peptides with an N-terminal 5-TAMRA (5-carboxytetramethylrhodamine) label were obtained from Proteogenix. The separate [Ca^2+^] conditions were mixed by an automated liquid handling robot (Hamilton MicroLab STARlet). 3 μL CaM (∼600 μm) was added to the first column of a 384-well plate (Corning) allowing for 24 dilutions of CaM (column-wise) at each of the four different free Ca^2+^ concentrations (row-wise). Meanwhile, the peptide concentration was kept constant (∼30 nm) in all wells. Calmodulin binding to either peptide was then monitored by measuring the fluorescence anisotropy (FA) signal of the 5-TAMRA-labeled peptide in a fluorescence plate reader (Spark Tecan, Zurich, Switzerland). For further details and graphical explanations of the assay, please refer to our previous work (55). The data from the resulting Ca^2+^-dependent calmodulin/EGL-19 or calmodulin/UNC-68-binding curves were fitted according to a stoichiometric equation:


$$\begin{align*} \Theta =&\ a\cdot \left(\frac{K_{\mathrm{D}}+{\left[\mathrm{Pep}\right]}_{\mathrm{tot}}+{\left[\mathrm{CaM}\right]}_{\mathrm{tot}}}{2\cdot{\left[\mathrm{Pep}\right]}_{\mathrm{tot}}}\right.\\ & \left. -\sqrt{{\left(\frac{K_{\mathrm{D}}+{\left[\mathrm{Pep}\right]}_{\mathrm{tot}}+{\left[\mathrm{CaM}\right]}_{\mathrm{tot}}}{2\cdot{\left[\mathrm{Pep}\right]}_{\mathrm{tot}}}\right)}^2-\frac{{\left[\mathrm{CaM}\right]}_{\mathrm{tot}}}{{\left[\mathrm{Pep}\right]}_{\mathrm{tot}}}}\right)+b \end{align*}$$

where *b* and *a* indicate the initial FA-plateau and the span in FA from low to high [CaM], respectively. [Pep]_tot_ and [CaM]_tot_ are the total concentrations of peptide and calmodulin, respectively, and *K*_D_ is the apparent calmodulin dissociation constant. Measurements were done in triplicate and curve fitting was done to the raw FA data using GraphPad Prism 8.4.2.

### Image analysis

All image analysis was performed in Fiji ImageJ (freely available from NIH). In images of morphology, brightness/contrast settings were gently adjusted without compromising the image data. Time-lapse acquisitions of motility were analyzed using the wrMTRCK plugin (56).

### Data analysis and statistics

Numeric data were collected in Microsoft Excel. Figure graphs and statistical tests were prepared using MathWorks MATLAB. All data were evaluated for normal distribution before selecting statistical analyses. Statistical significance is indicated as ^*^P < 0.05, ^*^^*^P < 0.01, ^*^^*^^*^P < 0.001. Where indicated these levels are Bonferroni-corrected. All statistical tests and *P*-values are given in Supplementary Material, Table S1. Figures were prepared in Inkscape.

## Supplementary Material

Supp_information_Heartworm_revised_2_ddad042

Supp_movie_S1_ddad042

Supp_movie_S2_ddad042

## Data Availability

All data is available upon request.

## References

[1] Clapham, D.E. (2007) Calcium signaling. Cell, 131, 1047–1058. 18083096 10.1016/j.cell.2007.11.028

[2] Berridge, M.J., Bootman, M.D. and Roderick, H.L. (2003) Calcium signalling: dynamics, homeostasis and remodelling. Nat. Rev. Mol. Cell Biol., 4, 517–529. 12838335 10.1038/nrm1155

[3] Jensen, H.H., Brohus, M., Nyegaard, M. and Overgaard, M.T. (2018) Human Calmodulin mutations. Front. Mol. Neurosci., 11. 10.3389/fnmol.2018.00396. PMC6243062 30483049

[4] Sorensen, A.B., Søndergaard, M.T. and Overgaard, M.T. (2013) Calmodulin in a heartbeat. FEBS J., 280, 5511–5532. 23663249 10.1111/febs.12337

[5] Halling, D.B., Liebeskind, B.J., Hall, A.W. and Aldrich, R.W. (2016) Conserved properties of individual Ca^2+^-binding sites in calmodulin. Proc. Natl. Acad. Sci., 113, E1216–E1225. 26884197 10.1073/pnas.1600385113 PMC4780646

[6] Nyegaard, M., Overgaard, M.T., Søndergaard, M.T., Vranas, M., Behr, E.R., Hildebrandt, L.L., Lund, J., Hedley, P.L., Camm, A.J., Wettrell, G. et al. (2012) Mutations in Calmodulin cause ventricular tachycardia and sudden cardiac death. Am. J. Hum. Genet., 91, 703–712. 23040497 10.1016/j.ajhg.2012.08.015 PMC3484646

[7] Crotti, L., Johnson, C.N., Graf, E., De Ferrari, G.M., Cuneo, B.F., Ovadia, M., Papagiannis, J., Feldkamp, M.D., Rathi, S.G., Kunic, J.D. et al. (2013) Calmodulin mutations associated with recurrent cardiac arrest in infants. Circulation, 127, 1009–1017. 23388215 10.1161/CIRCULATIONAHA.112.001216 PMC3834768

[8] Crotti, L., Spazzolini, C., Tester, D.J., Ghidoni, A., Baruteau, A., Beckmann, B., Behr, E.R., Bennett, J.S., Bezzina, C.R., Bhuiyan, Z.A. et al. (2019) Calmodulin mutations and life-threatening cardiac arrhythmias: insights from the international Calmodulinopathy registry. Eur. Heart J., 40, 2964–2975. 31170290 10.1093/eurheartj/ehz311 PMC6748747

[9] Brohus, M., Arsov, T., Wallace, D.A., Jensen, H.H., Nyegaard, M., Crotti, L., Adamski, M., Zhang, Y., Field, M.A., Athanasopoulos, V. et al. (2020) Infanticide vs. inherited cardiac arrhythmias. EP Eur., 23, 1–10. 10.1093/europace/euaa272 PMC7947592 33200177

[10] Nyegaard, M. and Overgaard, M.T. (2019) The international Calmodulinopathy registry: recording the diverse phenotypic spectrum of un-*CALM* hearts. Eur. Heart J., 40, 2976–2978. 31280324 10.1093/eurheartj/ehz463 PMC6748712

[11] Yamamoto, Y., Makiyama, T., Harita, T., Sasaki, K., Wuriyanghai, Y., Hayano, M., Nishiuchi, S., Kohjitani, H., Hirose, S., Chen, J. et al. (2017) Allele-specific ablation rescues electrophysiological abnormalities in a human iPS cell model of long-QT syndrome with a *CALM2* mutation. Hum. Mol. Genet., 26, 1670–1677. 28335032 10.1093/hmg/ddx073

[12] Limpitikul, W.B., Dick, I.E., Tester, D.J., Boczek, N.J., Limphong, P., Yang, W., Choi, M.H., Babich, J., DiSilvestre, D., Kanter, R.J. et al. (2017) A precision medicine approach to the rescue of function on malignant Calmodulinopathic long-QT syndrome. Circ. Res., 120, 39–48. 27765793 10.1161/CIRCRESAHA.116.309283 PMC5516949

[13] Søndergaard, M.T., Liu, Y., Brohus, M., Guo, W., Nani, A., Carvajal, C., Fill, M., Overgaard, M.T. and Chen, S.R.W. (2019) Diminished inhibition and facilitated activation of RyR2-mediated Ca^2+^ release is a common defect of arrhythmogenic calmodulin mutations. FEBS J., 286, 4554–4578. 31230402 10.1111/febs.14969

[14] Limpitikul, W.B., Dick, I.E., Joshi-Mukherjee, R., Overgaard, M.T., George, A.L. and Yue, D.T. (2014) Calmodulin mutations associated with long QT syndrome prevent inactivation of cardiac L-type Ca^2+^ currents and promote proarrhythmic behavior in ventricular myocytes. J. Mol. Cell. Cardiol., 74, 115–124. 24816216 10.1016/j.yjmcc.2014.04.022 PMC4262253

[15] Søndergaard, M.T., Sorensen, A.B., Skov, L.L., Kjaer-Sorensen, K., Bauer, M.C., Nyegaard, M., Linse, S., Oxvig, C. and Overgaard, M.T. (2015) Calmodulin mutations causing catecholaminergic polymorphic ventricular tachycardia confer opposing functional and biophysical molecular changes. FEBS J., 282, 803–816. 25557436 10.1111/febs.13184

[16] Berchtold, M.W., Zacharias, T., Kulej, K., Wang, K., Torggler, R., Jespersen, T., Chen, J.N., Larsen, M.R. and La Cour, J.M. (2016) The arrhythmogenic calmodulin mutation D129G dysregulates cell growth, calmodulin-dependent kinase II activity, and cardiac function in zebrafish. J. Biol. Chem., 291, 26636–26646. 27815504 10.1074/jbc.M116.758680 PMC5207174

[17] Tsai, W.-C., Guo, S., Olaopa, M.A., Field, L.J., Yang, J., Shen, C., Chang, C.-P., Chen, P.-S. and Rubart, M. (2020) Complex arrhythmia syndrome in a knock-in mouse model carrier of the N98S *Calm1* mutation. Circulation, 142, 1937–1955. 32929985 10.1161/CIRCULATIONAHA.120.046450 PMC7867118

[18] Corsi, A.K., Wightman, B. and Chalfie, M. (2015) A transparent window into biology: a primer on *Caenorhabditis elegans*. Genetics, 200, 387–407. 26088431 10.1534/genetics.115.176099 PMC4492366

[19] Engel, M.A., Wörmann, Y.R., Kaestner, H. and Schüler, C. (2022) An Optogenetic arrhythmia model—insertion of several Catecholaminergic polymorphic ventricular tachycardia mutations into *Caenorhabditis elegans* UNC-68 disturbs Calstabin-mediated stabilization of the ryanodine receptor homolog. Front. Physiol., 13. 10.3389/fphys.2022.691829. PMC8990320 35399287

[20] Fischer, E., Gottschalk, A. and Schüler, C. (2017) An optogenetic arrhythmia model to study catecholaminergic polymorphic ventricular tachycardia mutations. Sci. Rep., 7, 1–12. 29235522 10.1038/s41598-017-17819-8 PMC5727474

[21] Schüler, C., Fischer, E., Shaltiel, L., Steuer Costa, W. and Gottschalk, A. (2015) Arrhythmogenic effects of mutated L-type Ca^2+^-channels on an optogenetically paced muscular pump in *Caenorhabditis elegans*. Sci. Rep., 5, 14427. 26399900 10.1038/srep14427 PMC4585839

[22] Shtonda, B. and Avery, L. (2005) CCA-1, EGL-19 and EXP-2 currents shape action potentials in the *Caenorhabditis elegans* pharynx. J. Exp. Biol., 208, 2177–2190. 15914661 10.1242/jeb.01615 PMC1351090

[23] Avery, L. and You, Y. (2012) *C. elegans* feeding. WormBook, 1–23. 10.1895/wormbook.1.150.1 PMC3590810 22628186

[24] Laine, V., Segor, J.R., Zhan, H., Bessereau, J.-L. and Jospin, M. (2014) Hyperactivation of L-type voltage-gated Ca^2+^ channels in *Caenorhabditis elegans* striated muscle can result from point mutations in the IS6 or the IIIS4 segment of the 1 subunit. J. Exp. Biol., 217, 3805–3814. 25214488 10.1242/jeb.106732

[25] Kerr, R., Lev-Ram, V., Baird, G., Vincent, P., Tsien, R.Y. and Schafer, W.R. (2000) Optical imaging of calcium transients in neurons and pharyngeal muscle of *C. elegans*. Neuron, 26, 583–594. 10896155 10.1016/s0896-6273(00)81196-4

[26] Peters, M.A., Teramoto, T., White, J.Q., Iwasaki, K. and Jorgensen, E.M. (2007) A calcium wave mediated by gap junctions coordinates a rhythmic behavior in *C. elegans*. Curr. Biol., 17, 1601–1608. 17825560 10.1016/j.cub.2007.08.031

[27] Branicky, R. and Hekimi, S. (2006) What keeps *C. elegans* regular: the genetics of defecation. Trends Genet., 22, 571–579. 16911844 10.1016/j.tig.2006.08.006

[28] Allman, E., Waters, K., Ackroyd, S. and Nehrke, K. (2013) Analysis of Ca^2+^ Signaling motifs that regulate proton Signaling through the Na+/H^+^ exchanger NHX-7 during a rhythmic behavior in *Caenorhabditis elegans*. J. Biol. Chem., 288, 5886–5895. 23319594 10.1074/jbc.M112.434852 PMC3581405

[29] Dal Santo, P., Logan, M.A., Chisholm, A.D. and Jorgensen, E.M. (1999) The inositol trisphosphate receptor regulates a 50-second behavioral rhythm in *C. elegans*. Cell, 98, 757–767. 10499793 10.1016/s0092-8674(00)81510-x

[30] Jensen, H.H., Pedersen, G.A., Morgen, J.J., Parsons, M., Pedersen, S.F. and Nejsum, L.N. (2019) The Na^+^/H^+^ exchanger NHE1 localizes as clusters to cryptic lamellipodia and accelerates collective epithelial cell migration. J. Physiol., 597, 849–867. 30471113 10.1113/JP277383 PMC6355635

[31] Hums, I., Riedl, J., Mende, F., Kato, S., Kaplan, H.S., Latham, R., Sonntag, M., Traunmüller, L. and Zimmer, M. (2016) Regulation of two motor patterns enables the gradual adjustment of locomotion strategy in *Caenorhabditis elegans*. Elife, 5, 1–36. 10.7554/eLife.14116 PMC4880447 27222228

[32] Peliti, M., Chuang, J.S. and Shaham, S. (2013) Directional locomotion of *C. elegans* in the absence of external stimuli. PLoS One, 8, e78535. 24223821 10.1371/journal.pone.0078535 PMC3818405

[33] Vuong-Brender, T.T., Flynn, S., Vallis, Y. and de Bono, M. (2021) Neuronal calmodulin levels are controlled by CAMTA transcription factors. Elife, 10, 1–20. 10.7554/eLife.68238 PMC8428840 34499028

[34] Brini, M., Calì, T., Ottolini, D. and Carafoli, E. (2014) Neuronal calcium signaling: function and dysfunction. Cell. Mol. Life Sci., 71, 2787–2814. 24442513 10.1007/s00018-013-1550-7 PMC11113927

[35] Williams, S.N., Locke, C.J., Braden, A.L., Caldwell, K.A. and Caldwell, G.A. (2004) Epileptic-like convulsions associated with LIS-1 in the cytoskeletal control of neurotransmitter signaling in *Caenorhabditis elegans*. Hum. Mol. Genet., 13, 2043–2059. 15254012 10.1093/hmg/ddh209

[36] Mahoney, T.R., Luo, S. and Nonet, M.L. (2006) Analysis of synaptic transmission in *Caenorhabditis elegans* using an aldicarb-sensitivity assay. Nat. Protoc., 1, 1772–1777. 17487159 10.1038/nprot.2006.281

[37] Oh, K. and Kim, H. (2017) Aldicarb-induced paralysis assay to determine defects in synaptic transmission in *Caenorhabditis elegans*. BIO-PROTOCOL, 7. 10.21769/BioProtoc.2400. PMC5580937 28868330

[38] Graham, B., Shaw, M.-A. and Hope, I.A. (2020) Single amino acid changes in the ryanodine receptor in the human population have effects *in vivo* on *Caenorhabditis elegans* neuro-muscular function. Front. Genet., 11, 1–16. 32174957 10.3389/fgene.2020.00037 PMC7054344

[39] Yan, Z., Cheng, X., Li, Y., Su, Z., Zhou, Y. and Liu, J. (2022) Sexually dimorphic neurotransmitter release at the neuromuscular junction in adult *Caenorhabditis elegans*. Front. Mol. Neurosci., 14, 1–11. 10.3389/fnmol.2021.780396 PMC8841764 35173578

[40] Gottschalk, A., Almedom, R.B., Schedletzky, T., Anderson, S.D., Yates, J.R. and Schafer, W.R. (2005) Identification and characterization of novel nicotinic receptor-associated proteins in *Caenorhabditis elegans*. EMBO J., 24, 2566–2578. 15990870 10.1038/sj.emboj.7600741 PMC1176467

[41] Torayama, I., Ishihara, T. and Katsura, I. (2007) *Caenorhabditis elegans* integrates the signals of butanone and food to enhance chemotaxis to butanone. J. Neurosci., 27, 741–750. 17251413 10.1523/JNEUROSCI.4312-06.2007 PMC6672901

[42] Karabinos, A., Büssing, I., Schulze, E., Wang, J., Weber, K. and Schnabel, R. (2003) Functional analysis of the single calmodulin gene in the nematode *Caenorhabditis elegans* by RNA interference and 4-D microscopy. Eur. J. Cell Biol., 82, 557–563. 14703012 10.1078/0171-9335-00347

[43] VanBerkum, M.F.A. and Goodman, C.S. (1995) Targeted disruption of Ca^2+^-calmodulin signaling in *drosophila* growth cones leads to stalls in axon extension and errors in axon guidance. Neuron, 14, 43–56. 7826640 10.1016/0896-6273(95)90239-2

[44] Bae, B., Gruner, H.N., Lynch, M., Feng, T., So, K., Oliver, D., Mastick, G.S., Yan, W., Pieraut, S. and Miura, P. (2020) Elimination of *Calm1* long 3′-UTR mRNA isoform by CRISPR–Cas9 gene editing impairs dorsal root ganglion development and hippocampal neuron activation in mice. RNA, 26, 1414–1430. 32522888 10.1261/rna.076430.120 PMC7491327

[45] Yasuda, R., Hayashi, Y. and Hell, J.W. (2022) CaMKII: a central molecular organizer of synaptic plasticity, learning and memory. Nat. Rev. Neurosci., 23, 666–682. 36056211 10.1038/s41583-022-00624-2

[46] Hwang, H.S., Nitu, F.R., Yang, Y., Walweel, K., Pereira, L., Johnson, C.N., Faggioni, M., Chazin, W.J., Laver, D., George, A.L. et al. (2014) Divergent regulation of ryanodine receptor 2 calcium release channels by arrhythmogenic human calmodulin missense mutants. Circ. Res., 114, 1114–1124. 24563457 10.1161/CIRCRESAHA.114.303391 PMC3990285

[47] Prakash, O., Gupta, N., Milburn, A., McCormick, L., Deugi, V., Fisch, P., Wyles, J., Thomas, N.L., Antonyuk, S., Dart, C. et al. (2022) Calmodulin variant E140G associated with long QT syndrome impairs CaMKIIδ autophosphorylation and L-type calcium channel (Cav1.2) inactivation. J. Biol. Chem., 307, 102777. 10.1016/j.jbc.2022.102777 PMC9830374 36496072

[48] Li, Q., Marcu, D.-C., Palazzo, O., Turner, F., King, D., Spires-Jones, T.L., Stefan, M.I. and Busch, K.E. (2020) High neural activity accelerates the decline of cognitive plasticity with age in *Caenorhabditis elegans*. Elife, 9, 1–35. 10.7554/eLife.59711 PMC7685709 33228848

[49] Wes, P.D. and Bargmann, C.I. (2001) *C. elegans* odour discrimination requires asymmetric diversity in olfactory neurons. Nature, 410, 698–701. 11287957 10.1038/35070581

[50] de Bono, M., Tobin, D.M., Davis, M.W., Avery, L. and Bargmann, C.I. (2002) Social feeding in *Caenorhabditis elegans* is induced by neurons that detect aversive stimuli. Nature, 419, 899–903. 12410303 10.1038/nature01169 PMC3955269

[51] Dwyer, D.S., Awatramani, P., Thakur, R., Seeni, R. and Aamodt, E.J. (2015) Social feeding in *Caenorhabditis elegans* is modulated by antipsychotic drugs and calmodulin and may serve as a protophenotype for asociality. Neuropharmacology, 92, 56–62. 25576370 10.1016/j.neuropharm.2014.12.027

[52] Sharp, P.M. and Bradnam, K.R. (1997) In Riddle, D.L., Blumenthal, T., Meyer, B.J. and Priess, J.R. (eds), C. elegans II: Appendix 3 Codon Usage in C. elegans, 2nd edn. Cold Spring Harbor Laboratory Press, Cold Spring Harbor, NY.

[53] Arribere, J.A., Bell, R.T., Fu, B.X.H., Artiles, K.L., Hartman, P.S. and Fire, A.Z. (2014) Efficient marker-free recovery of custom genetic modifications with CRISPR/Cas9 in *Caenorhabditis elegans*. Genetics, 198, 837–846. 25161212 10.1534/genetics.114.169730 PMC4224173

[54] Paix, A., Folkmann, A., Rasoloson, D. and Seydoux, G. (2015) High efficiency, homology-directed genome editing in *Caenorhabditis elegans* using CRISPR-Cas9ribonucleoprotein complexes. Genetics, 201, 47–54. 26187122 10.1534/genetics.115.179382 PMC4566275

[55] Brohus, M., Søndergaard, M.T., Wayne Chen, S.R., van Petegem, F. and Overgaard, M.T. (2019) Ca^2+^-dependent calmodulin binding to cardiac ryanodine receptor (RyR2) calmodulin-binding domains. Biochem. J., 476, 193–209. 30530841 10.1042/BCJ20180545 PMC6340113

[56] Nussbaum-Krammer, C.I., Neto, M.F., Brielmann, R.M., Pedersen, J.S. and Morimoto, R.I. (2015) Investigating the spreading and toxicity of prion-like proteins using the metazoan model Organism *C. elegans*. J. Vis. Exp., 95, e52321. 10.3791/52321 PMC4354510 25591151

[57] Vassilakopoulou, V., Calver, B.L., Thanassoulas, A., Beck, K., Hu, H., Buntwal, L., Smith, A., Theodoridou, M., Kashir, J., Blayney, L. et al. (2015) Distinctive malfunctions of calmodulin mutations associated with heart RyR2-mediated arrhythmic disease. Biochim. Biophys. Acta Gen. Subj., 1850, 2168–2176. 10.1016/j.bbagen.2015.07.001 26164367

[58] Søndergaard, M.T., Tian, X., Liu, Y., Wang, R., Chazin, W.J., Chen, S.R.W. and Overgaard, M.T. (2015) Arrhythmogenic calmodulin mutations affect the activation and termination of cardiac ryanodine receptor-mediated Ca^2+^ release. J. Biol. Chem., 290, 26151–26162. 26309258 10.1074/jbc.M115.676627 PMC4646266

[59] Wang, K., Brohus, M., Holt, C., Overgaard, M.T., Wimmer, R. and Van Petegem, F. (2020) Arrhythmia mutations in calmodulin can disrupt cooperativity of Ca^2+^ binding and cause misfolding. J. Physiol., 598, 1169–1186. 32012279 10.1113/JP279307

[60] Søndergaard, M.T., Liu, Y., Guo, W., Wei, J., Wang, R., Brohus, M., Overgaard, M.T. and Chen, S.R.W. (2020) Role of cardiac ryanodine receptor calmodulin-binding domains in mediating the action of arrhythmogenic calmodulin N-domain mutation N54I. FEBS J., 287, 2256–2280. 31763755 10.1111/febs.15147

[61] Wang, K., Holt, C., Lu, J., Brohus, M., Larsen, K.T., Overgaard, M.T., Wimmer, R. and Van Petegem, F. (2018) Arrhythmia mutations in calmodulin cause conformational changes that affect interactions with the cardiac voltage-gated calcium channel. Proc. Natl. Acad. Sci., 115, E10556–E10565. 30348784 10.1073/pnas.1808733115 PMC6233071

[62] Jiménez-Jáimez, J., Doza, J.P., Ortega, Á., Macías-Ruiz, R., Perin, F., Rodríguez-Vázquez Del Rey, M.M., Ortiz-Genga, M., Monserrat, L., Barriales-Villa, R., Blanca, E. et al. (2016) Calmodulin 2 mutation N98S is associated with unexplained cardiac arrest in infants due to low clinical penetrance electrical disorders. PLoS One, 11, 1–10. 10.1371/journal.pone.0153851 PMC4839566 27100291

[63] Makita, N., Yagihara, N., Crotti, L., Johnson, C.N., Beckmann, B.M., Roh, M.S., Shigemizu, D., Lichtner, P., Ishikawa, T., Aiba, T. et al. (2014) Novel calmodulin mutations associated with congenital arrhythmia susceptibility. Circ. Cardiovasc. Genet., 7, 466–474. 24917665 10.1161/CIRCGENETICS.113.000459 PMC4140998

[64] Fujita, S., Nakagawa, R., Futatani, T., Igarashi, N., Fuchigami, T., Saito, S., Ohno, S., Horie, M. and Hatasaki, K. (2019) Long QT syndrome with a de novo *CALM2* mutation in a 4-year-old boy. Pediatr. Int., 61, 852–858. 31283864 10.1111/ped.13959

