# Supplementary material for: Human calmodulin mutations cause arrhythmia and affect neuronal function in C. elegans
Source: Hum Mol Genet. 2023 Mar 15;32(12):2068–83. doi: 10.1093/hmg/ddad042 (PMC10244212; doi:10.1093/hmg/ddad042)
Supplement: Supp_information_Heartworm_revised_2_ddad042 [file supp_information_heartworm_revised_2_ddad042.pdf]

Supplementary information to

"Human calmodulin mutations cause arrhythmia and affect neuronal function in *C. elegans*"

Helene H Jensen[1], Magnus T Frantzen[1], Jonas L Wesseltoft[1], Ana-Octavia Busuioc[1], Katrine V Møller[1], Malene Brohus[1], Palle R Duun[2], Mette Nyegaard[2], Michael T Overgaard[1], Anders Olsen[1,*]

[1]Department of Chemistry and Bioscience, Aalborg University, 9220 Aalborg Ø, Denmark
[2]Department of Health Science and Technology, Aalborg University, 9220 Aalborg Ø, Denmark

*Correspondence: Anders Olsen, ao@bio.aau.dk, +45 3069 8155

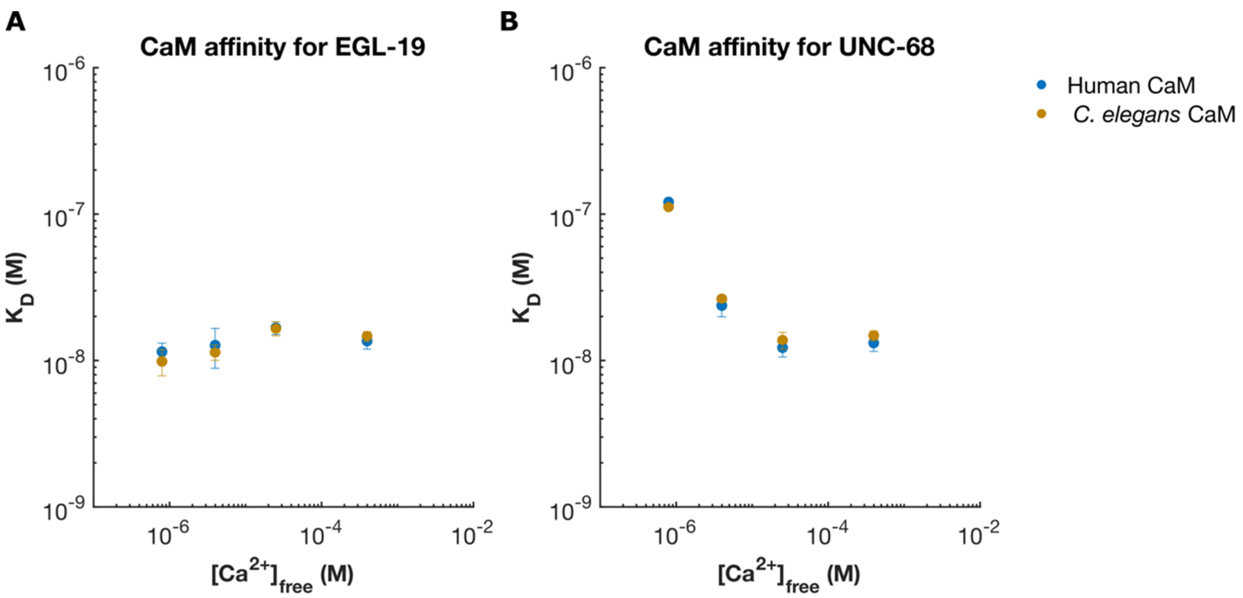

Supplementary figure S1: No difference in the Ca^{2+} dependent binding of CaM to EGL-19 and UNC-68. Binding affinity (K_D) of purified calmodulin (CaM) to the predicted calmodulin binding domains of EGL-19 (orthologue of $Ca_V1.2$) and UNC-68 (orthologue of RyR2) was measured in a well-established fluorescence anisotropy assay (1,2).

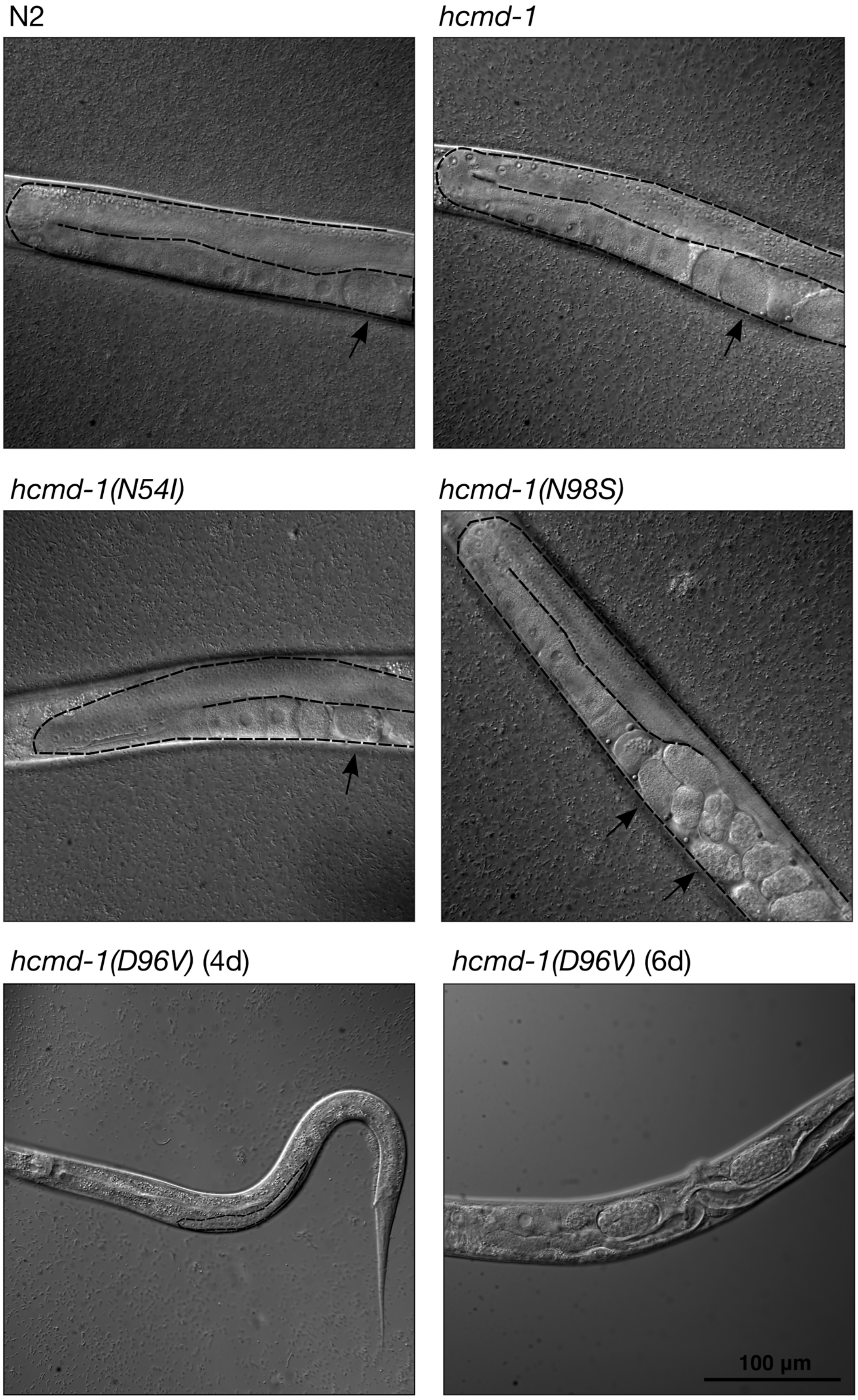

Supplementary figure S2: Bright-field images of germlines and fertilized eggs in adult *hcmd-1* worms. Adult *C. elegans* (4d for all and 6d for *hmd-1(D96V)* as indicated) were imaged using DIC microscopy. The germline is indicated with dotted lines. Wild-type animals have two hairpin shaped germline tubes. The eggs develop as they move through the tubes. Almost-mature oocytes have a square shape with an evident nucleus. Arrows indicate examples of mature eggs. For *hcmd-1(N98S)*, mature eggs and developing embryos accumulated in the worms. In most *hcmd-1(N98S)* worms, embryos hatched inside the worms in late adulthood, causing bagging and killing the mother. At day 4, the

germline in *hmd-1(D96V)* was not fully developed. At day 6, the germline of *hcmd-1(D96V)* mutants was under proliferated and contained few germ cells and oocytes.

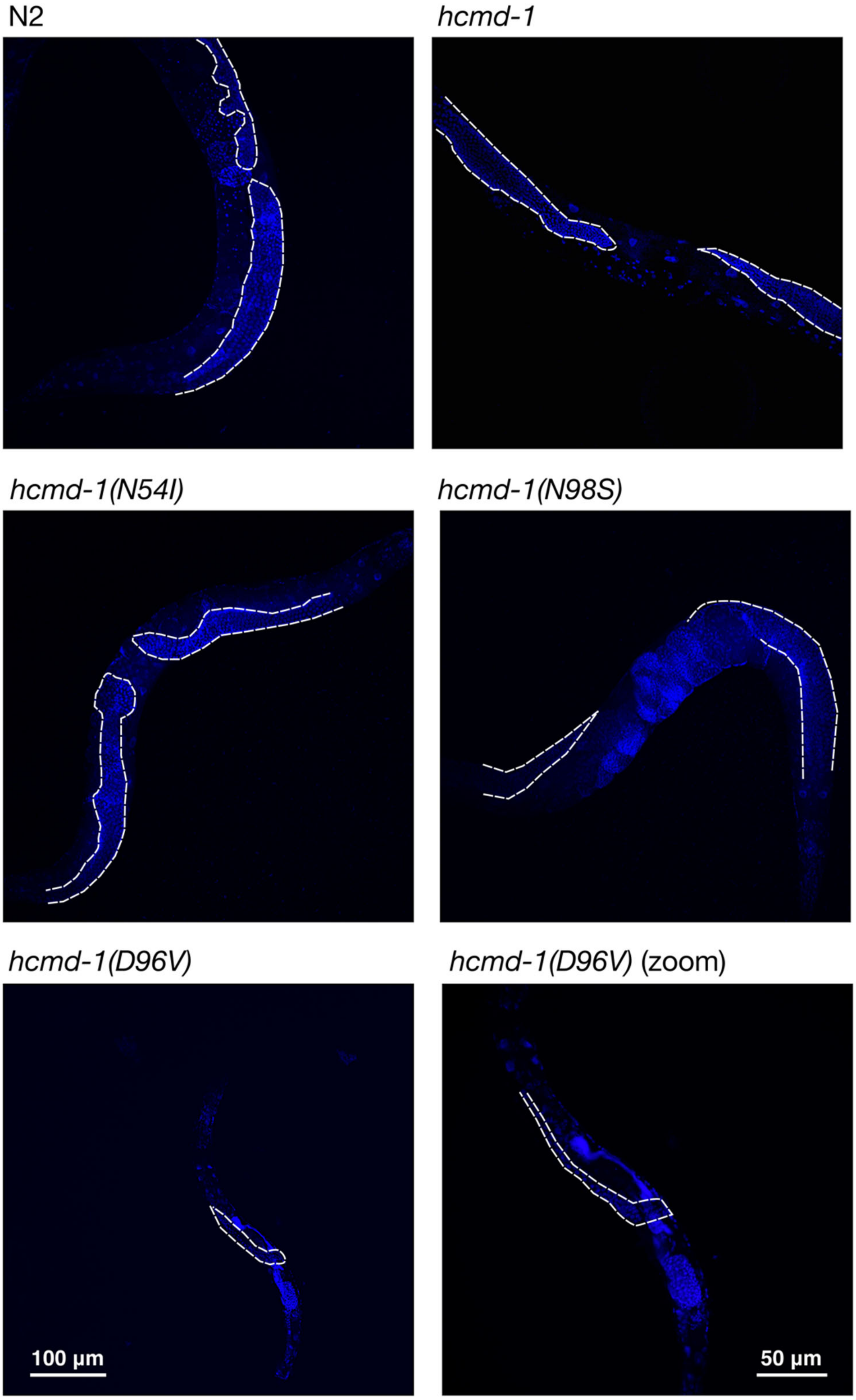

Supplementary figure S3: Germline in adult *hcmd-1* worms visualized with DAPI stain. Adult *C. elegans* were stained with DAPI to visualize cell nuclei or nuclei under development (blue). Germlines are indicated with white dotted lines.

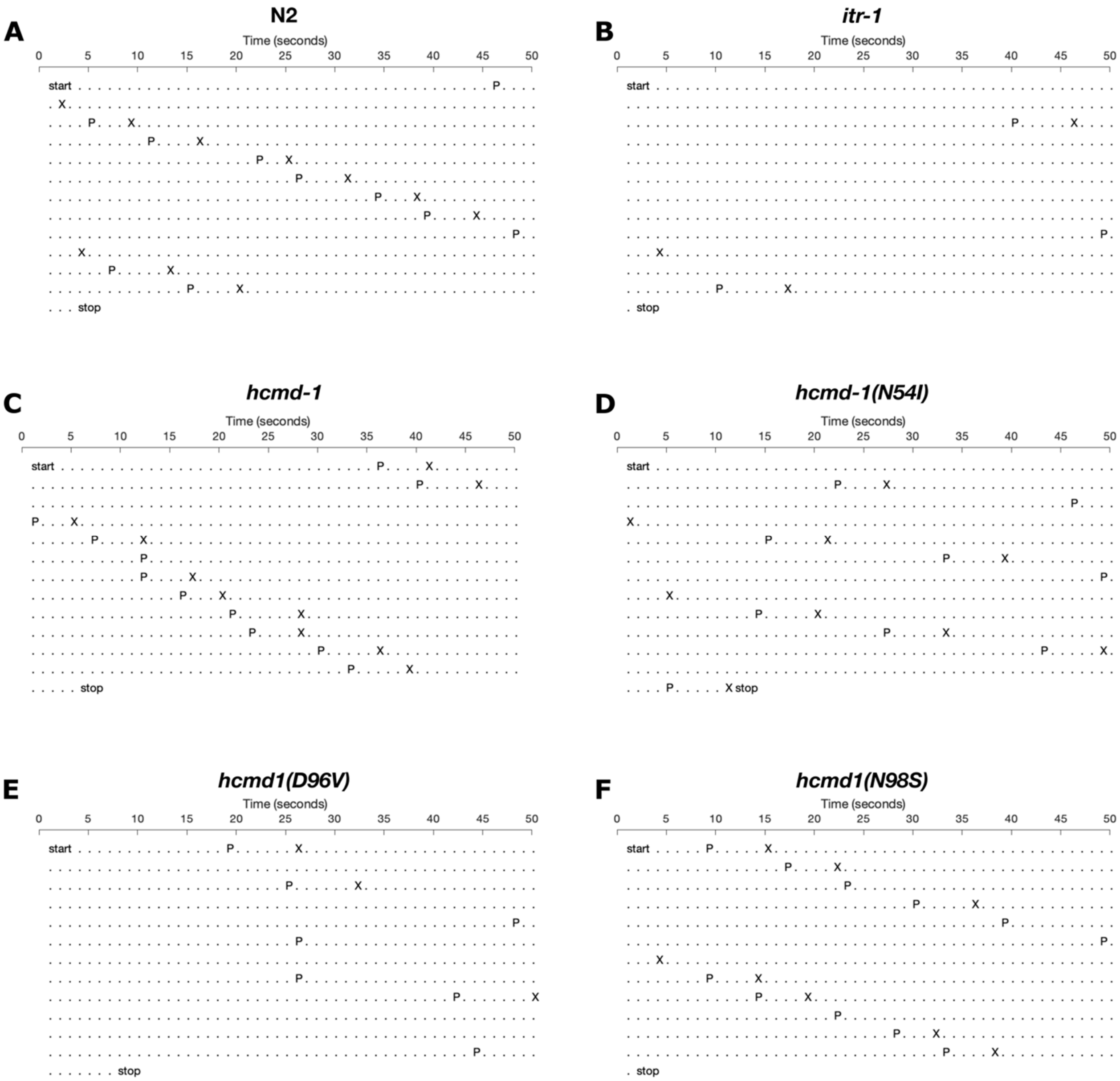

Supplementary figure S4. Example ethograms of DMP for all mutants. Each worm was observed while freely moving on OP50 bacteria for 10 min or until moving away from the bacterial lawn. pBoc (P) or Exp (X) were manually registered.

Supplementary movie S1. Muscle convulsions in *hcmd-1(D96V)*. Example movie of movement of a *hcmd-1(D96V)* worm with a platinum picker.

Supplementary movie S2. No muscle convulsions in *hcmd-1*. Example movie of movement of a *hcmd-1* worm with a platinum picker.

Supplementary table S1: p-values for all statistical tests.

Sample 1	Sample 2	p-value	Significance
**Figure 1**			
**C. N-lobe Ca^{2+} binding**			
Student's t-test			
Human CaM	*C. elegans* CaM	0.74	NS
**C. C-lobe Ca^{2+} binding**			
Student's t-test			
Human CaM	*C. elegans* CaM	0.79	NS
**Figure 2**			
**B. Timeline of sizes**			
Two-way ANOVA with Tukey-Kramer post-hoc test			
N2	*hcmd-1*	0.0288	*
hcmd-1	*hcmd-1(N54I)*	<0.00001	***
hcmd-1	*hcmd-1(D96V)*	<0.00001	***
hcmd-1	*hcmd-1(N98S)*	0.6723	NS
**C: Timeline of viable offspring**			
Repeated measures ANOVA and Tukey-Kramer post-hoc test			
N2	*hcmd-1*	0.9300	NS
hcmd-1	*hcmd-1(N54I)*	1.000	NS
hcmd-1	*hcmd-1(D96V)*	0.0002842	***
hcmd-1	*hcmd-1(N98S)*	0.9995	NS
**D: Sum of laid eggs**			
Bonferroni-corrected Student's t-test. Corrected significance level: 0.0125			
N2	*hcmd-1*	0.03920	NS
hcmd-1	*hcmd-1(N54I)*	0.5441	NS
hcmd-1	*hcmd-1(D96V)*	<0.00001	***
hcmd-1	*hcmd-1(N98S)*	0.4956	NS
**Figure 3**			
**A: Pharynx pump frequency**			
Bonferroni-corrected Wilcoxon rank sum test. Corrected significance level: 0.0100			
N2	*egl-19*	<0.00001	***
N2	*hcmd-1*	0.2879	NS
hcmd-1	*hcmd-1(N54I)*	0.0011	*
hcmd-1	*hcmd-1(D96V)*	<0.00001	***
hcmd-1	*hcmd-1(N98S)*	0.6220	NS
**B: pBoc per 10 min**			
Bonferroni-corrected Wilcoxon rank sum test. Corrected significance level: 0.0100			
N2	*itr-1*	<0.00001	***
N2	*hcmd-1*	0.0071	*

hcmd-1	*hcmd-1(N54I)*	$4.155*10^{-4}$	**
hcmd-1	*hcmd-1(D96V)*	<0.00001	***
hcmd-1	*hcmd-1(N98S)*	0.1333	NS
**C: Exp/pBoc ratio**			
Bonferroni-corrected Wilcoxon rank sum test. Corrected significance level: 0.0100			
N2	*itr-1*	0.2389	NS
N2	*hcmd-1*	0.0166	NS
hcmd-1	*hcmd-1(N54I)*	$4.815*10^{-4}$	**
hcmd-1	*hcmd-1(D96V)*	$2.420*10^{-4}$	**
hcmd-1	*hcmd-1(N98S)*	0.1534	NS
**D: pBoc-to-Exp time (s)**			
Bonferroni-corrected Student's t-test. Corrected significance level: 0.0100			
N2	*itr-1*	<0.00001	***
N2	*hcmd-1*	0.7627	NS
hcmd-1	*hcmd-1(N54I)*	0.0067	*
hcmd-1	*hcmd-1(D96V)*	$5.482*10^{-4}$	**
hcmd-1	*hcmd-1(N98S)*	0.6446	NS
**Figure 4**			
**A: Thrashing**			
Bonferroni-corrected Student's t-test. Corrected significance level: 0.0100			
N2	*unc-68*	<0.00001	***
N2	*hcmd-1*	0.0561	NS
hcmd-1	*hcmd-1(N54I)*	<0.00001	***
hcmd-1	*hcmd-1(D96V)*	0.0039	*
hcmd-1	*hcmd-1(N98S)*	0.0104	NS
**B: Maximum crawling speed**			
Bonferroni-corrected Student's t-test. Corrected significance level: 0.0100			
N2	*unc-68*	<0.00001	***
N2	*hcmd-1*	0.0150	NS
hcmd-1	*hcmd-1(N54I)*	0.0920	NS
hcmd-1	*hcmd-1(D96V)*	0.4104	NS
hcmd-1	*hcmd-1(N98S)*	0.7713	NS
**C: Track length per 30 s (speed)**			
Bonferroni-corrected Wilcoxon rank sum test. Corrected significance level: 0.0100			
N2	*unc-68*	<0.00001	***
N2	*hcmd-1*	0.1320	NS
hcmd-1	*hcmd-1(N54I)*	0.7442	NS
hcmd-1	*hcmd-1(D96V)*	0.0932	NS
hcmd-1	*hcmd-1(N98S)*	0.1703	NS
**D: Distance traveled**			
Bonferroni-corrected Wilcoxon rank sum test. Corrected significance level: 0.0100			
N2	*unc-68*	<0.00001	***

N2	*hcmd-1*	0.7768	NS
hcmd-1	*hcmd-1(N54I)*	0.8109	NS
hcmd-1	*hcmd-1(D96V)*	$3.986*10^{-4}$	**
hcmd-1	*hcmd-1(N98S)*	0.4454	NS
**E: Directionality**			
Bonferroni-corrected Wilcoxon rank sum test. Corrected significance level: 0.0100			
N2	*unc-68*	0.1467	NS
N2	*hcmd-1*	0.0016	**
hcmd-1	*hcmd-1(N54I)*	0.0059	*
hcmd-1	*hcmd-1(D96V)*	<0.00001	***
hcmd-1	*hcmd-1(N98S)*	0.9525	NS
**Figure 5**			
**A: Muscle cramps upon being moved**			
Bonferroni-corrected Fisher's Exact test. Corrected significance level: 0.0125			
N2	*hcmd-1*	1	NS
hcmd-1	*hcmd-1(N54I)*	1	NS
hcmd-1	*hcmd-1(D96V)*	$3.705*10^{-14}$	***
hcmd-1	*hcmd-1(N98S)*	1	NS
**B: Aldicarb paralysis**			
Bonferroni-corrected log rank test. Corrected significance level: 0.0083			
N2	*egl-19*	0.003298	*
N2	*unc-68*	<0.00001	***
N2	*hcmd-1*	0.1875	NS
hcmd-1	*hcmd-1(N54I)*	<0.00001	***
hcmd-1	*hcmd-1(D96V)*	<0.00001	***
hcmd-1	*hcmd-1(N98S)*	0.00085	**
**B: Levamisole paralysis**			
Bonferroni-corrected log rank test. Corrected significance level: 0.0083			
N2	*egl-19*	<0.00001	***
N2	*unc-68*	<0.00001	***
N2	*hcmd-1*	0.1405	NS
hcmd-1	*hcmd-1(N54I)*	<0.00001	***
hcmd-1	*hcmd-1(D96V)*	<0.00001	***
hcmd-1	*hcmd-1(N98S)*	0.4861	NS
**C: Chemotaxis**			
Bonferroni-corrected Wilcoxon rank sum test. Corrected significance level: 0.0125			
N2	*hcmd-1*	<0.00001	***
hcmd-1	*hcmd-1(N54I)*	0.0435	NS
hcmd-1	*hcmd-1(D96V)*	0.4559	NS
hcmd-1	*hcmd-1(N98S)*	0.0106	*

Supplementary table S2: CRISPR guides and repair templates

	crRNA (5'-3')	ssODN (5'-3')	Used for strain
dpy-10 (cn64)	GCUACCAUAGGC ACCACGAG	CACTTGAACTTCAATACGGCAAGATGAGAATGACTGGAAACCGTACC GCATGCGGTGCCTATGGTAGCGGAGCTTCACATGGCTTCAGACCAAC AGCCTAT	
F100Y	GGACGGAAAUGG CUUCAUCU	TCCTATTTGCAGGTTTTCGACAAGGACGGAAATGGCTATATTTCTGCA GCTGAACTGCGCCACGTCATGACCAACTTGGG	*hcmd-1* (OLS160)
T114Q T148A	GAUUUACUUGGU UGUCAUCA	ATCAAATTTCCCATATTTCAGAGTTCGTCCAGATGATGACTGCTAAATA AATCACACCAATCGGGATCTGAAGCCGCCGT	*hcmd-1* (OLS160)
N54I	UCAGGACAUGAU CAACGAAG	GGGAAATCGATGGTTCCGTTTCCGTCAGCGTCAACCTCGATAATCATA TCTTGAAGCTCGGCTTCAGTCGGATTTTGTCC	*hcmd-1(N54I)* OLS178
N98S	UUGCAGGUUUUC GACAAGGA	GTCATGACGTGGCGCAGTTCAGCTGCAGAGATGTAACCGCTACCATCC TTGTCGAAAACCTGCAAATAGGAAGATTTGAA	*hcmd-1(N98S)* OLS183
D96V		GACGTGGCGCAGTTCAGCTGCAGAAATATAGCCGTTACCAACTTTGTC AAAGACCTGCAAATAGGAAGATTTGAATGAGA	*hcmd-1(D96V)* OLS171

Supplementary table S3: Concentration of CRISPR components

Stock concentration	
tracrRNA	200µM
crRNA	100µM
ssODN	100µM*

* except for *dpy-10* which was 10µM

Supplementary table S4: PCR primers

Name	Sequence	Used for strain	Annealing temp. used	Reference
hcmd-1 F100Y mut fw	5'-CGGAAATGGCTATATTTCTGC-3'	OLS160	58.5ºC	
hcmd-1 F100Y wt fw	5'-AAGGACGGAAATGGCTTCAT-3'	OLS160	58.5ºC	
hcmd-1 F100Y rv	5'-ATATCGGCTTCACGGATCAT-3'	OLS160	58.5ºC	
hcmd-1 T144Q T148A fw	5'-GGAGAGAAGCTAACGGACGA-3'	OLS160	55.0ºC	
hcmd-1 T144Q T148A mut rv	5'-TTTAGCAGTCATCATCTGGACG-3'	OLS160	55.0ºC	
hcmd-1 T144Q T148A wt rv	5'- TACTTGGTTGTCATCATGGTGA-3'	OLS160	55.0ºC	
hcmd-1 N54I mut fw	5'-CAAGATATGATTATCGAGGTTG-3'	OLS178	53.0ºC	
hcmd-1 N54I wt fw	5'-TCAGGACATGATCAACGAAGT-3'	OLS178	53.0ºC	
hcmd-1 N54I rv	5'-TTGTCGAAAACCTGCAAATAG-3'	OLS178	53.0ºC	
hcmd-1 D96V mut fw	5'- CTTTGACAAAGTTGGTAACGGC-3'	OLS183	56.0ºC	
hcmd-1 D96V wt fw	5'-TTTCGACAAGGACGGAAATG-3'	OLS183	56.0ºC	
hcmd-1 D96V rv	5'-TCACCCTCATAATTGACTTGTCC-3'	OLS183	56.0ºC	
hcmd-1 N98S mut fw	5'-GGATGGTAGCGGTTACATCTCT-3'	OLS171	55.0ºC	
hcmd-1 N98S wt fw	5'-GGACGGAAATGGCTATATTTCT-3'	OLS171	55.0ºC	
hcmd-1 N98S rv	5'-GCAGTCATCATCTGGACGAAC-3'	OLS171	55.0ºC	
oEF60 (fw)	5'-GTACTTGAACCTAGGTAC-'3	TR2171	45.4ºC	Fischer et al 2017 (3)
oEF61 (fw)	5'-GTGTACTCTGTTTCGTC-3'	TR2171	49.2ºC	Fischer et al 2017 (3)
oEF59 (rv)	5'-CTCAGCGGTGGAAAC-3'	TR2171		Fischer et al 2017 (3)

References to Supplementary information

1. Brohus, M., Arsov, T., Wallace, D.A., Jensen, H.H., Nyegaard, M., Crotti, L., Adamski, M., Zhang, Y., Field, M.A., Athanasopoulos, V., *et al.* (2020) Infanticide vs. inherited cardiac arrhythmias. *EP Eur.*, **23**, 1–10.
2. Brohus, M., Søndergaard, M.T., Wayne Chen, S.R., van Petegem, F. and Overgaard, M.T. (2019) Ca^{2+}-dependent calmodulin binding to cardiac ryanodine receptor (RyR2) calmodulin-binding domains. *Biochem. J.*, **476**, 193–209.
3. Fischer, E., Gottschalk, A. and Schüler, C. (2017) An optogenetic arrhythmia model to study catecholaminergic polymorphic ventricular tachycardia mutations. *Sci. Rep.*, **7**, 1–12.
